# Development and Laboratory Evaluation of a *porA*-Targeting TaqMan qPCR Assay for *Neisseria gonorrhoeae*: Clinical Specimen Validation and Molecular Estimation Using Cultured Reference Strains

**DOI:** 10.3390/ijms27167335

**Published:** 2026-08-17

**Authors:** Changmi Oh, Jin-Ho Kim

**Affiliations:** 1 Department of Medical Laser, College of Medicine, Dankook University, Cheonan 31116, Republic of Korea; o72260499@dankook.ac.kr; 2 Biomedical Research Institute, Dankook University Hospital, Cheonan 31116, Republic of Korea

**Keywords:** CFU/mL-equivalent molecular estimation, clinical specimen validation, molecular diagnosis, *Neisseria gonorrhoeae*, *porA* pseudogene, sexually transmitted infection, TaqMan qPCR

## Abstract

*Neisseria gonorrhoeae* is a major sexually transmitted pathogen and global public health concern. A *porA* pseudogene-targeting TaqMan quantitative PCR (qPCR) assay was evaluated as a complementary laboratory-based method for the qualitative detection of *N. gonorrhoeae* and exploratory Z017-calibrated molecular estimation in cultured reference strains. Two candidate probe sets were evaluated, and the probe set generating a 157 bp amplicon was selected for a defined two-step qPCR workflow with a 52 min on-instrument amplification runtime. In silico analysis showed compatible target site arrangements in all 201 *N. gonorrhoeae* genomes and none in 114 non-target organisms. The assay detected *N. gonorrhoeae* DNA down to 1 × 10^−5^ ng/µL, with no amplification of the tested non-target organisms. All 23 *N. gonorrhoeae* reference strains and 80 *N. gonorrhoeae*-positive clinical specimens were detected, whereas no amplification was observed in the 20 negative clinical specimens, using DNA extracted from the primary clinical specimens. A standard curve was generated from the titered Z017 reference material (R^2^ = 0.999; efficiency, 94.6%), which enabled reproducible qPCR-derived CFU/mL-equivalent molecular estimation in cultured World Health Organization reference strains. The qPCR-derived estimates were lower than the paired culture-derived CFU/mL values, and the paired log10-transformed values did not show a statistically significant correlation. These findings provide an assay design and validation-oriented laboratory-based framework combining in silico sequence assessment, the qualitative detection of *N. gonorrhoeae* in clinical specimens, and exploratory molecular estimation in cultured reference strains, supporting future assay development and bacterial burden research.

## 1. Introduction

*Neisseria gonorrhoeae* is a major sexually transmitted bacterial pathogen and global public health concern [1,2]. The World Health Organization (WHO) estimated approximately 82.4 million new gonococcal infections among adults aged 15–49 years in 2020, with over 1 million curable sexually transmitted infections being acquired every day worldwide [1,3]. Gonococcal infections are frequently asymptomatic, particularly in women [4]. Untreated infections can lead to adverse outcomes, including pelvic inflammatory disease, infertility, adverse pregnancy outcomes, neonatal infections, and an increased risk of human immunodeficiency virus acquisition [5]. The global emergence of antimicrobial-resistant *N. gonorrhoeae* has increased the need for accurate and timely laboratory diagnosis, ongoing surveillance, and improved molecular tools for infection monitoring [6,7].

Nucleic acid amplification tests (NAATs) are widely used in the laboratory-based diagnosis of *N. gonorrhoeae* because of their high analytical sensitivity, specificity, and operational convenience [8,9]. Current clinical NAATs are well suited for qualitative detection, particularly in urine and genital swab specimens, and they have become a central component of gonorrhea diagnostics [10,11]. Although routine diagnostic NAAT workflows are primarily designed and reported for qualitative classification, molecular measurements that provide calibrated target DNA estimates may offer complementary information for laboratory-based research and assay development applications [12]. Additionally, culture remains an essential method for phenotypic antimicrobial susceptibility testing, the investigation of suspected treatment failure, and the surveillance of emerging resistance [13]. Consequently, molecular assays that provide calibrated target DNA estimates may complement routine qualitative detection and offer additional value when interpreted as complementary molecular estimates rather than direct measures of viable bacterial burden. Such assays may also be used as adjunctive tools rather than replacements for established NAATs or culture-based antimicrobial susceptibility testing [14,15].

*N. gonorrhoeae* remains a substantial global health burden, which is further compounded by the emergence of antimicrobial resistance [16,17]. Molecular detection is additionally complicated by the close genetic relatedness between pathogenic and commensal *Neisseria* species [11,15,18]. Cross-reactivity with non-gonococcal *Neisseria* species and sequence variations in target regions can result in false-positive or false-negative findings when molecular targets are not carefully selected [18]. The *porA* pseudogene has been identified as a valuable gonococcus-associated molecular target [19]. Feavers and Maiden first described the gonococcal *porA* pseudogene and its evolutionary relevance, and subsequent studies demonstrated that *porA* pseudogene-targeting real-time PCR assays can provide sensitive and specific detection of *N. gonorrhoeae* across different clinical specimen types and validation settings [20,21,22,23]. Building on this established foundation, the present study focused on the design and integrated validation of a new *porA*-targeting probe set across in silico sequence assessment, multiple gonococcal reference strain collections, clinical urine and vaginal swab specimens, and Z017-based exploratory molecular estimation in cultured WHO reference strains. This framework extends the application of the established *porA* target by combining sequence-based evaluation, qualitative detection, and culture–molecular comparison within a single assay development study.

Quantitative real-time PCR has the potential to extend qualitative pathogen detection by enabling the estimation of target DNA burden [24]. Such information may be useful during assay development and in future studies investigating bacterial burdens, specimen types, treatment responses, and infection dynamics [14,24]. Nevertheless, qPCR-based molecular estimation of bacterial burden requires careful interpretation because culture-derived colony-forming units (CFUs) reflect viable and culturable organisms, whereas qPCR detects target DNA regardless of bacterial viability, including DNA from damaged or non-viable bacteria [25,26]. In this context, a paired comparison between qPCR-derived estimates and culture-derived CFU/mL values can provide complementary information on the relationship and method-dependent differences between molecular and culture-based measurements. Accordingly, qPCR-derived estimates should be regarded as standard-curve-based molecular estimates that may reflect relative target DNA burden under the tested conditions, rather than direct equivalents of culture-derived viable CFUs [27].

In this study, a *porA*-targeting TaqMan qPCR assay was evaluated as a complementary laboratory-based method for the qualitative detection of *N. gonorrhoeae*. The selected probe set was additionally assessed through in silico inclusivity and exclusivity analyses to evaluate sequence-level coverage and target discrimination. A conventional two-step TaqMan qPCR workflow was empirically selected under the defined laboratory conditions and evaluated for analytical specificity, the lowest observed detectable input concentration in the original serial dilution experiment, and qualitative detection performance using the WHO *N. gonorrhoeae* reference strains provided by the National Collection of Type Cultures (NCTC), American Type Culture Collection (ATCC), and National Culture Collection for Pathogens (NCCP). The clinical qualitative detection performance of the assay was assessed using DNA extracted from primary *N. gonorrhoeae*-positive urine and vaginal swab specimens, as well as negative clinical controls. A standard curve generated from titered *N. gonorrhoeae* Z017 reference material was used to provide a Z017-calibrated exploratory molecular estimation framework for the cultured WHO reference strains. The resulting values were interpreted as standard-curve-based CFU/mL-equivalent molecular estimates rather than direct measures of viable bacterial counts. Accordingly, the quantitative component focused on characterizing Z017-calibrated molecular estimates in cultured reference strains in relation to paired culture-derived CFU/mL values, thereby providing a basis for future evaluation of quantitative applications in clinical specimens.

## 2. Results

### 2.1. Selection of the porA-Targeting Probe Set

Two candidate probe sets targeting the *porA* pseudogene of *N. gonorrhoeae* were designed and evaluated for qPCR assay development. The detailed oligonucleotide sequences, amplicon sizes, and preliminary screening results are provided in Supplementary Table S1.

Both candidate sets generated amplification signals under the tested conditions. However, probe set 1 showed lower Ct values and a higher fluorescence intensity than probe set 2 during preliminary screening. Therefore, probe set 1 was selected for subsequent in silico sequence assessment, empirical assay condition selection, analytical evaluation, clinical specimen testing, and Z017-calibrated exploratory molecular estimation in the cultured WHO reference strains.

### 2.2. In Silico Inclusivity and Exclusivity of the Selected porA-Targeting Probe Set

The selected probe set was evaluated against 201 *N. gonorrhoeae* genome accessions, all of which retained the forward primer–probe–reverse primer arrangement required for the expected target amplicon. Complete sequence identity for all three oligonucleotides was observed in 200 accessions; in CP048250.1, the forward primer showed 95% sequence identity, whereas the probe and reverse primer each showed 100% identity. Accession-level sequence match results are provided in Supplementary Table S2.

The exclusivity analysis included 114 non-target organisms comprising bacterial, fungal, protozoal, and viral taxa. None showed a compatible arrangement of primer-binding sites or an intervening probe-binding site capable of generating the expected amplicon. Organism-level oligonucleotide match and amplicon assessment results are provided in Supplementary Table S3.

### 2.3. Evaluation of the Two-Step TaqMan qPCR Assay

The selected probe set was further evaluated through stepwise empirical testing for two-step TaqMan qPCR using DNA extracted from *N. gonorrhoeae* ATCC 19424. Because the assay development experiments were not designed as a full factorial optimization study, the final reaction conditions were selected empirically based on amplification performance under the examined conditions.

The final cycling conditions comprised an annealing/extension step at 60 °C, and the final reaction mixture contained 500 nM each of the forward and reverse primers and 1000 nM TaqMan probe. Among the probe concentrations evaluated, 1000 nM produced the lowest Ct value and highest fluorescence signal and was therefore selected for the final assay conditions (Table 1).

**Table 1:** Stepwise empirical evaluation of qPCR reaction conditions for the selected *porA* probe set.

Evaluated Parameter	Template	Tested Condition	Ct Value	RFU Value	Result
Annealing/extension temperature	ATCC 19424 DNA	54 °C	21.59	9000	Positive
		56 °C	21.56	9750	Positive
		58 °C	21.56	10,500	Positive
		**60** **°C**	**21.54**	**12,200**	**Positive**
		62 °C	22.01	10,500	Positive
		64 °C	22.17	8500	Positive
Probe concentration	ATCC 19424 DNA	500 nM	21.02	11,600	Positive
		**1000 nM**	**20.91**	**15,200**	**Positive**
		2000 nM	20.93	15,000	Positive
No-template control		2000 nM	None	Baseline or none	Negative

**Figure 1:**
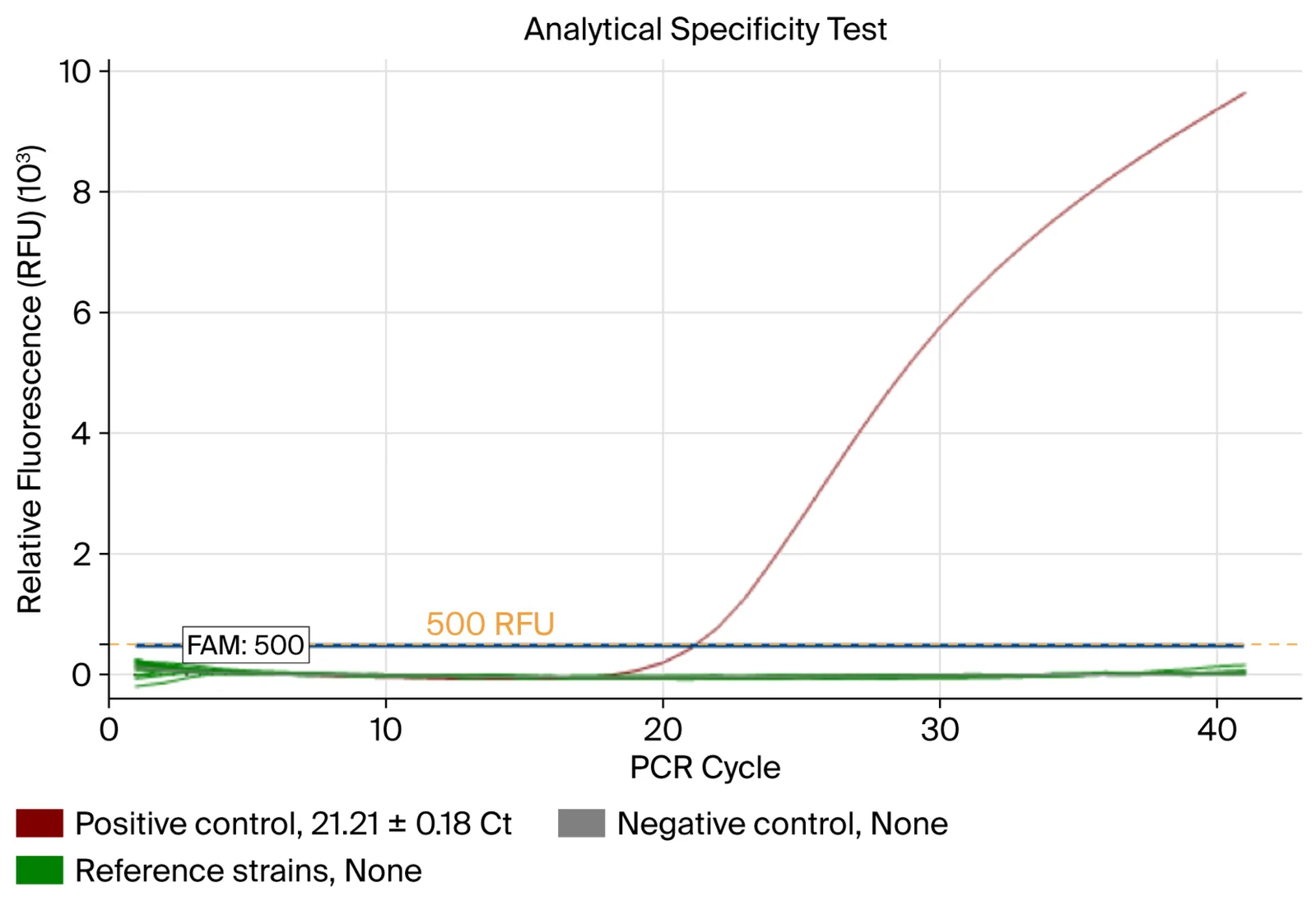
Analytical specificity of the *porA*-targeting TaqMan qPCR assay for *Neisseria gonorrhoeae*. Each target was tested in three technical replicates, and representative amplification curves are shown. A fixed fluorescence threshold of 500 RFU was used for the analysis, as indicated by the blue and orange lines. The plots were exported using the same display range and plotting settings as those used for Figure 2. Positive control: *N. gonorrhoeae* ATCC 19424; reference strains: *N. oralis*, *N. dentiae*, *N. shayeganii*, *N. flavescens*, *N.* sp., *N. yagbaofengii*, *N. meningitidis*, *N.* sp. *KCOM 1440*, *N. flava MA1-1*, *N. subflava MA3-1*, and *C. trachomatis* serovar E; negative control: no-template (nuclease-free water).

**Figure 2:**
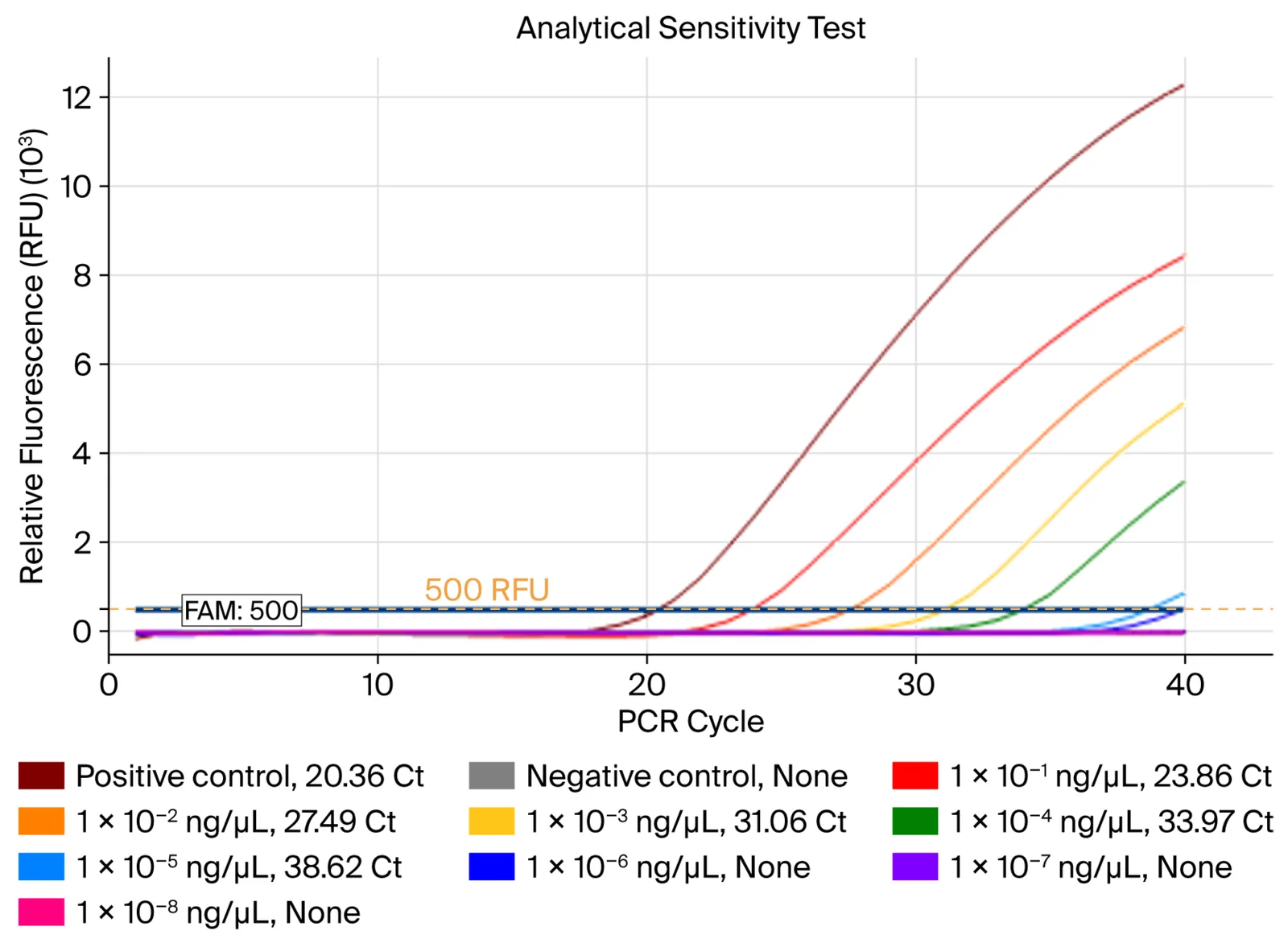
Analytical sensitivity test of the *porA*-targeting TaqMan qPCR assay for *Neisseria gonorrhoeae*. Each target was tested in three technical replicates, and representative amplification curves are shown. A fixed fluorescence threshold of 500 RFU was used for the analysis, as indicated by the blue and orange lines. The plots were exported using the same display range and plotting settings as those used for Figure 1. Positive control: ATCC 19424; serially diluted ATCC 19424 from 1 × 10^−1^ ng/µL to 1 × 10^−8^ ng/µL for sensitivity testing; negative control: no-template (nuclease-free water).

Conditions were selected through sequential screening on the basis of the Ct value, fluorescence intensity, amplification curve morphology, and absence of amplification in the no-template control. The final selected conditions are indicated in **bold**. Ct and RFU values were obtained during the probe concentration evaluation and describe the empirical condition selection process. Because Figure 1 and Figure 2 are generated in separate qPCR runs for analytical specificity and sensitivity testing, respectively, their absolute RFU values are not used for direct comparisons with those reported in Table 1.

The final two-step qPCR protocol consisted of initial denaturation at 95 °C for 3 min, followed by 40 cycles at 95 °C for 5 s and 60 °C for 30 s. The total amplification runtime was 52 min. This value represents the on-instrument qPCR amplification runtime only and does not include specimen collection, DNA extraction, reaction setup, or post-run data interpretation. No amplification was observed in the no-template control under the final assay conditions.

### 2.4. Analytical Specificity and Sensitivity

Analytical specificity was performed using DNA extracted from non-gonococcal *Neisseria* species and *Chlamydia trachomatis* serovar E. Each target was tested in technical triplicate. Amplification was observed in the *N. gonorrhoeae*-positive control but not in any of the other samples. The positive control showed a mean Ct value of 21.21 ± 0.18 across the three technical replicates. No amplification was observed in the tested non-target organisms or no-template control (Figure 1).

To assess the lowest observed detectable concentration in the serial dilution experiment, 10-fold serial dilutions of *N. gonorrhoeae* ATCC 19424 DNA, ranging from 1.0 to 1 × 10^−8^ ng/µL, were tested. Each dilution was tested in technical triplicate. Using a fixed fluorescence threshold of 500 relative fluorescence units (RFU), the lowest DNA concentration that produced a typical sigmoidal amplification curve and crossed the threshold within 40 cycles was 1 × 10^−5^ ng/µL. The corresponding Ct value was 38.62 ± 0.04. No amplification was observed at lower concentrations under the tested conditions (Figure 2).

### 2.5. Validation of the Final Assay Conditions Using N. gonorrhoeae Reference Strains

The final assay conditions were evaluated using *N. gonorrhoeae* reference strains, including the WHO (NCTC), ATCC, and NCCP strains. The assay detected all 23 tested *N. gonorrhoeae* reference strains, namely, 12 WHO (NCTC), 4 ATCC, and 7 NCCP strains (Table 2). All tested *N. gonorrhoeae* reference strains produced amplification curves that successfully crossed the 500-RFU fluorescence threshold before 40 cycles. No amplification was observed in the no-template control. The corresponding amplification curves, including the no-template control, are presented in Supplementary Figure S2.

### 2.6. Validation Using Directly Extracted Clinical Specimens

The final *porA*-targeting qPCR assay conditions were evaluated using DNA extracted from 100 clinical specimens. The validation set comprised 40 *N. gonorrhoeae*-positive urine specimens, 40 *N. gonorrhoeae*-positive vaginal swab specimens, 10 negative urine specimens, and 10 negative vaginal swab specimens. Although purified DNA was used as the qPCR template, the DNA was directly extracted from urine and vaginal swab specimens.

Among the 40 *N. gonorrhoeae*-positive urine specimens and 40 *N. gonorrhoeae*-positive vaginal swab specimens, all were detected as positive using the developed qPCR assay, corresponding to a detection rate of 100% (Supplementary Table S4).

Among the 10 negative urine specimens and 10 negative vaginal swab specimens, none showed amplification within 40 cycles. The overall positive detection rate was 100% (80/80), and no amplification was observed in the 20 negative clinical specimens (Supplementary Table S4).

### 2.7. Supportive Retrospective Qualitative Agreement

A separate retrospective urine specimen subset with available routine Cobas 4800 CT/NG results was analyzed for a supportive qualitative agreement assessment. This subset was independent of the original 100-specimen clinical validation set and included 30 Cobas 4800 *N. gonorrhoeae*-positive urine specimens and 40 Cobas 4800-negative urine specimens. The developed *porA*-targeting qPCR assay showed an overall percent agreement of 100% (70/70), a positive percent agreement of 100% (30/30), and a negative percent agreement of 100% (40/40) relative to Cobas 4800 CT/NG testing. Cohen’s kappa was 1.00. No discordant results were observed in this retrospective urine specimen subset (Table 3; Supplementary Table S5).

### 2.8. Generation of the Z017-Based Standard Curve

**Figure 3:**
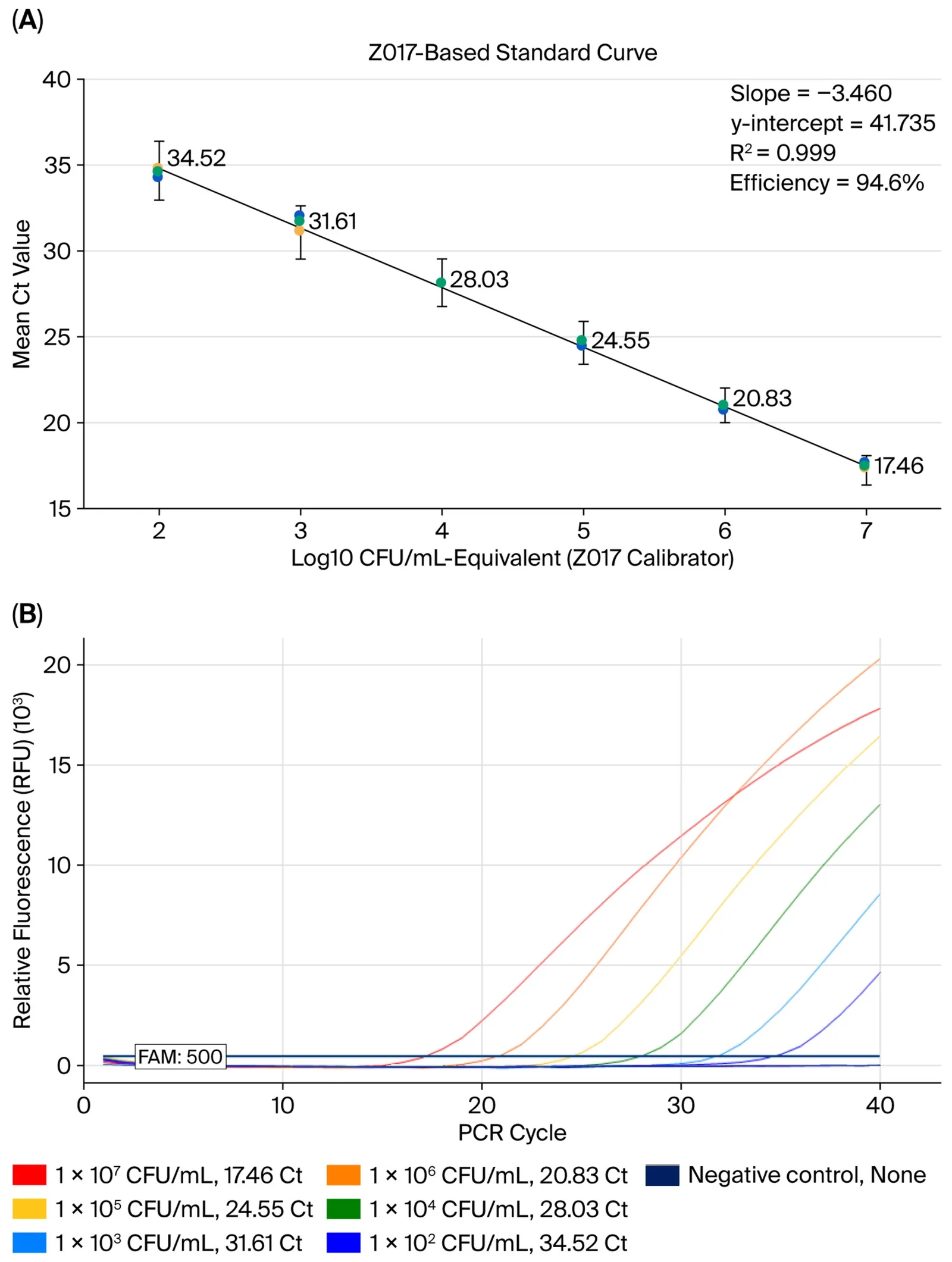
Z017-based standard curve and representative amplification curves for the *porA*-targeting TaqMan qPCR assay. (**A**) Standard curve generated from triplicate qPCR measurements of serially diluted *N. gonorrhoeae* Z017 DNA. Each point represents the mean Ct value, and error bars indicate the standard deviation of three technical replicates; R^2^ = 0.999, slope = −3.460, intercept = 41.735, and efficiency = 94.6%. Individual Ct values and summary statistics are provided in Supplementary Table S6. (**B**) Representative amplification curves obtained from serially diluted titered *N. gonorrhoeae* Z017 reference material. A fixed fluorescence threshold of 500 RFU was used for the analysis, as indicated by the blue line. Positive control: ATCC 19424; serially diluted Z017 from 1 × 10^7^ CFU/mL to 1 × 10^1^ CFU/mL for Z017-based standard curve; negative control: no-template (nuclease-free water).

A standard curve was generated using titered *N. gonorrhoeae* Z017 reference material (ZeptoMetrix, Buffalo, NY, USA). The extracted Z017 DNA was serially diluted to generate a standard curve, with points corresponding to concentrations ranging from 1.00 × 10^7^ to 1.00 × 10^2^ CFU/mL-equivalent concentrations. Each Z017-calibrated concentration was analyzed in triplicate using the final two-step qPCR assay conditions. For each standard concentration, the mean Ct value and standard deviation (SD) were calculated from triplicate measurements. The standard curve was computed using the mean Ct values, and SD error bars were generated (Figure 3A). Individual Ct values and summary statistics for each standard concentration are provided in Supplementary Table S6.

The standard curve showed a coefficient of determination (R^2^) of 0.999 across the evaluated concentration range. The slope and intercept were found to be –3.460 and 41.735, respectively, and the calculated amplification efficiency was 94.6% (Figure 3). The no-template control did not cross the 500-RFU fluorescence threshold within 40 cycles (Figure 3B).

Ct values were also compared between two Z017 preparation workflows: DNA extraction, followed by the serial dilution of the extracted DNA, and the serial dilution of the titered Z017 material, followed by DNA extraction from each dilution. The mean Ct difference between the two workflows was 0.36 cycles.

### 2.9. qPCR-Derived Molecular Estimation of Cultured WHO Reference Strains

The Z017-derived standard curve was used to estimate the qPCR-derived CFU/mL-equivalent concentrations of 12 cultured WHO *N. gonorrhoeae* reference strains. For each strain, DNA was extracted from the broth culture and analyzed in triplicate using the final qPCR assay conditions. The mean Ct value obtained from the triplicate measurements for each reference strain was interpolated against the Z017-derived standard curve to calculate a qPCR-derived CFU/mL-equivalent estimate.

Across the 12 cultured WHO reference strains, the mean Ct values ranged from 19.24 to 22.65. The qPCR-derived estimates ranged from 3.28 × 10^5^ to 3.18 × 10^6^ CFU/mL-equivalent. The coefficient of variation ranged from 0.05 to 3.22% (Table 4). For every cultured WHO reference strain, the culture-derived CFU/mL value exceeded the corresponding qPCR-derived CFU/mL-equivalent estimate. The culture/qPCR ratio ranged from 3.59 to 370.98, and the geometric mean culture/qPCR ratio was 15.35:1.

The log10-transformed qPCR-derived CFU/mL-equivalent estimates were not significantly correlated with the paired culture-derived CFU/mL values (Pearson’s r = −0.456, *p* = 0.136). The mean log10 difference, calculated as log10(qPCR-derived CFU/mL-equivalent estimate) − log10(culture-derived CFU/mL), was −1.19 log10 CFU/mL. This corresponded to a geometric mean culture/qPCR ratio of 15.35:1 (Supplementary Figure S3).

## 3. Discussion

In this study, a *porA*-targeting TaqMan qPCR assay was evaluated as a complementary laboratory-based approach for the qualitative detection of *N. gonorrhoeae* in reference strains and clinical urine and vaginal swab specimens. The final assay targeted a 157 bp region of the *porA* pseudogene and used a two-step qPCR workflow with a 52 min amplification runtime. In silico analysis further showed that the selected probe set retained the expected target amplicon arrangement across all 201 evaluated *N. gonorrhoeae* accessions, whereas no compatible amplicon-forming arrangement was identified among the 114 non-target organisms. Under the tested conditions, the assay showed a lowest observed detectable *N. gonorrhoeae* DNA concentration of 1 × 10^−5^ ng/µL, showed no amplification in the tested non-gonococcal organisms, detected all evaluated *N. gonorrhoeae* reference strains, and successfully detected all *N. gonorrhoeae*-positive urine and vaginal swab specimens in the clinical validation set. In addition, the Z017-derived standard curve was applied to the cultured WHO reference strains to generate exploratory qPCR-derived CFU/mL-equivalent estimates.

The *porA* pseudogene is an established molecular target of *N. gonorrhoeae* [19]. Feavers and Maiden first described the evolutionary relevance of the gonococcal *porA* pseudogene, and Hjelmevoll et al. subsequently reported a *porA* pseudogene-targeting real-time PCR assay with a detection limit of less than 7.5 genome equivalents per reaction [20,21]. Whiley et al. evaluated a *porA* pseudogene-targeting real-time PCR assay using genital and extragenital clinical specimens, and Hjelmevoll et al. subsequently validated a *porA*-targeting real-time PCR assay against culture techniques [22,23]. More recently, Aitlhaj-Mhand et al. evaluated an in-house *porA*-targeting real-time PCR assay using anal swab specimens and a commercial NAAT as the comparator [28]. These studies further support the utility of the established *porA* target across different specimen populations and clinical validation designs. The present study integrated in silico assessment of a newly designed probe set with laboratory-based evaluation of a two-step TaqMan qPCR workflow using clinical urine and vaginal swab specimens; WHO (NCTC), ATCC, and NCCP reference strains; and titered Z017 reference material for exploratory molecular estimations in cultured WHO reference strains. These elements extend the application of the established *porA* target through a distinct assay design and validation framework encompassing sequence coverage, multiple reference strain collections, two clinical specimen types, and a paired culture–molecular comparison. Because previously reported assays differed in oligonucleotide design, specimen type, reaction conditions, and validation scope, the present results provide complementary evidence rather than a direct performance ranking among *porA*-targeting assays.

The lowest DNA input detected in the original serial dilution experiment was 0.01 pg per reaction. Aitlhaj-Mhand et al. previously reported the detection of 0.6 pg of *N. gonorrhoeae* DNA using a *porA* pseudogene-targeting real-time PCR assay, whereas Hjelmevoll et al. reported a detection limit of less than 7.5 genome equivalents per reaction [21,28]. Using an approximate *N. gonorrhoeae* genome size of 2.2 Mb and estimated genome mass of approximately 2.4 fg, the 0.01 pg input detected in the present study theoretically corresponds to approximately 4.1 genome equivalents per reaction. Ahmadi et al. reported reproducible detection at 72 copies per reaction using a LAMP assay [29]. In contrast, Chen et al. reported a detection limit of 1.1 × 10^4^–1.8 × 10^4^ CFU/mL for a protein-based ELISA method [30]. Because these studies differed in reference materials, extraction procedures, template input volumes, reaction chemistries, instrument platforms, positivity criteria, replicate designs, and reporting units, these values are provided as only descriptive literature context and should not be interpreted as direct analytical sensitivity comparisons or as evidence of the superior performance of the assay presented herein (Supplementary Table S7).

The 52 min runtime represents the on-instrument qPCR amplification program and does not include DNA extraction, specimen preparation, reaction setup, or result reporting. Therefore, it should be interpreted as an operational characteristic of the present laboratory-based workflow rather than as a complete sample-to-answer time or evidence of superior overall performance relative to assays evaluated under different experimental conditions. The analytical specificity results showed no amplification in the tested non-gonococcal *Neisseria* species or *C. trachomatis*. These laboratory findings are consistent with the in silico exclusivity analysis, in which none of the 114 non-target organisms showed a compatible primer–probe arrangement capable of generating the expected amplicon. These findings are noteworthy because the molecular detection of *N. gonorrhoeae* can be affected by the close genetic relatedness of pathogenic and commensal *Neisseria* species, as well as sequence-related limitations in NAAT target design [18,31].

The assay also detected all 23 tested *N. gonorrhoeae* reference strains, including the WHO (NCTC), ATCC, and NCCP strains. Validation across multiple reference strains is an important analytical step because *N. gonorrhoeae* exhibits genetic diversity, and assay performance cannot be adequately represented by a single laboratory strain alone [32]. The detection of all the strains within the evaluated reference panel, together with the in silico inclusivity results across 201 *N. gonorrhoeae* genome accessions, supports qualitative detection across the tested strain collection and sequence dataset; further evaluation using broader collections of circulating variants would extend the assessment of strain coverage.

Clinical specimen testing showed that the assay classified 40/40 *N. gonorrhoeae*-positive urine and 40/40 *N. gonorrhoeae*-positive vaginal swab specimens as positive, while no amplification was observed in the 10 negative urine and 10 negative vaginal swab controls. The clinical specimen evaluation focused on the qualitative classification of directly extracted urine and vaginal swab specimens. Chen et al. reported a direct qPCR workflow for quantifying *N. gonorrhoeae* growth in liquid media and testing phenotypic antimicrobial susceptibility, including testing in the presence of urogenital flora and clinical swab eluent [33]. Although the study addressed a different experimental objective and used a different sample-processing workflow and reporting framework, it highlighted the importance of evaluating qPCR performance in clinically relevant specimen-associated matrices. In this context, the present evaluation using directly extracted urine and vaginal swab specimens provides complementary evidence for assay performance across two clinically relevant urogenital matrices.

In addition to the original clinical validation set, a separate retrospective urine specimen subset with available routine Cobas 4800 CT/NG results was evaluated for supportive qualitative agreement. This subset comprised 30 Cobas 4800 *N. gonorrhoeae*-positive urine specimens and 40 Cobas 4800-negative urine specimens. The developed assay showed an overall percent agreement of 100% (70/70), a positive percent agreement of 100% (30/30), and a negative percent agreement of 100% (40/40) relative to Cobas 4800 CT/NG testing. Cohen’s kappa coefficient was 1.00. No discordant results were observed (Table 3; Supplementary Table S5). These findings support qualitative concordance between the two assays within the retrospectively matched subset and complement the results obtained from the original clinical validation set. Formal evaluation of clinical equivalence, non-inferiority, or superiority would require a dedicated prospective comparator design.

DNA was extracted directly from the original clinical specimens rather than from residual DNA extracts generated by a commercial platform. This design enabled the evaluation of the assay using DNA obtained from primary urine and vaginal swab specimens within the study workflow. Because neither a dedicated inhibitor study nor an internal amplification control was included, the potential influence of urine- or vaginal swab-associated inhibition remains to be characterized in future studies. The inclusion of urine and vaginal swab specimens enabled assessments across two clinically relevant urogenital specimen matrices that are commonly used for NAAT-based gonorrhea testing [9,34]. The present findings therefore provide qualitative detection data across both specimen types, while direct between-matrix comparisons and matrix-specific clinical performance characteristics remain subjects for further evaluation.

Titered *N. gonorrhoeae* Z017 reference material was used to generate a standard curve for qPCR-derived CFU/mL-equivalent estimation. The Z017-derived standard curve showed linearity across the tested range, with an R^2^ value of 0.999 and an amplification efficiency of 94.6%. A comparison of the two preparation workflows (DNA extraction followed by serial dilution and the serial dilution of the titered material followed by DNA extraction) showed a mean Ct difference of 0.36 cycles. The Z017-derived standard curve was subsequently applied to cultured WHO reference strains to generate qPCR-derived CFU/mL-equivalent estimates.

A comparison between the Z017-calibrated qPCR-derived CFU/mL-equivalent estimates and paired culture-derived CFU/mL values showed a consistent directional difference between the two measurements. For all 12 cultured WHO reference strains, the qPCR-derived CFU/mL-equivalent estimate was lower than the corresponding culture-derived CFU/mL value. The culture/qPCR ratios ranged from 3.59 to 370.98, with a geometric mean ratio of 15.35:1. However, the log10-transformed qPCR-derived estimates were not significantly correlated with the paired culture-derived CFU/mL values (Pearson’s r = −0.456, *p* = 0.136). Accordingly, the paired measurements showed a consistent direction of difference, although the magnitude varied substantially among strains, whereas their strain-level relationship was not statistically significant under the present conditions. The mean log10 difference, calculated as log10(qPCR-derived CFU/mL-equivalent estimate) − log10(culture-derived CFU/mL), was −1.19 log10 CFU/mL.

This discrepancy should be interpreted as a descriptive method-dependent difference rather than as evidence of direct interchangeability or a conversion factor applicable to other strains, specimen types, or laboratories. Culture-based CFU enumeration reflects viable and culturable organisms, whereas qPCR detects target DNA and may also reflect DNA from damaged, non-culturable, or non-viable organisms [26,35]. In addition, the observed difference may have been influenced by Z017 reference material calibration, DNA extraction recovery, volume conversion, and the distinct measurement principles of qPCR and colony enumeration. This interpretation is also consistent with the principle that DNA-based amplification methods do not inherently distinguish DNA derived from viable and non-viable bacteria without additional viability discrimination approaches, such as EMA- or PMA-based treatment [36,37,38]. The low intra-assay variability observed across the triplicate qPCR measurements indicates reproducible estimation within the tested cultured reference strain workflow. This quantitative component provides a reproducible framework for Z017-calibrated molecular estimation in cultured WHO reference strains and may support future studies extending this approach to clinical bacterial burden assessment and the interpretation of molecular load values in clinical specimens.

The intended role of the developed assay is to complement established diagnostic and surveillance approaches. Commercial NAATs remain central to the routine qualitative detection of *N. gonorrhoeae*, whereas culture remains necessary for phenotypic antimicrobial susceptibility testing, the investigation of suspected treatment failure, and antimicrobial resistance surveillance [35,39]. Accordingly, this study provides a complementary laboratory-based workflow that combines *porA*-targeting qualitative detection in directly extracted clinical urine and vaginal swab specimens with exploratory standard-curve-based molecular estimation in cultured WHO reference strains.

Recent molecular diagnostic approaches for *N. gonorrhoeae* have expanded beyond identifying organisms to incorporating the detection of antimicrobial resistance-associated determinants. Recent assays have enabled the simultaneous detection of *N. gonorrhoeae* and ciprofloxacin resistance, rapid screening of the ceftriaxone resistance-associated penA-60 allele, and direct identification of penA and 23S rRNA mutations associated with decreased cephalosporin susceptibility and azithromycin resistance in clinical specimens [40,41,42]. Although the present *porA*-targeting assay was designed for species-level detection rather than antimicrobial resistance prediction, these advances highlight the complementary potential of integrating pathogen identification with resistance-associated molecular markers.

This study had several limitations, which should be interpreted in relation to the intended scope of the assay. Although DNA was directly extracted from primary urine and vaginal swab specimens, the assay did not include an internal amplification control or a dedicated inhibitor assessment experiment. Therefore, matrix-associated inhibition was not independently characterized in individual specimens, particularly those with low target abundance or atypical amplification profiles. Future clinical studies should incorporate an endogenous or exogenous internal amplification control and evaluate extraction recovery and matrix-associated inhibition using representative urine and vaginal swab specimens. In addition, serial dilutions near the positivity threshold were not repeatedly tested. Therefore, the lowest concentration detected in the original serial dilution experiment should be interpreted as the lowest observed detectable concentration under the tested conditions.

The clinical specimens were tested once per sample; therefore, future repeat-testing studies could further evaluate reproducibility in clinical matrices. The negative clinical panel was limited to 10 urine and 10 vaginal swab specimens and did not include a broader range of specimens from individuals with other sexually transmitted infections or commensal *Neisseria* carriage. Although the in silico exclusivity analysis across 114 non-target organisms provided complementary sequence-level evidence supporting target discrimination, larger prospective studies with more diverse negative clinical panels would further characterize clinical specificity and potential cross-reactivity under routine testing conditions. The Cobas 4800 CT/NG comparison was restricted to a retrospectively traceable matched subset and was intended as a qualitative agreement analysis rather than an independent clinical performance evaluation or demonstration of equivalence to the commercial comparator.

Additionally, bacterial load estimation was performed using cultured WHO reference strains and was not directly applied to clinical specimens. Because the residual clinical specimens were used only for qualitative assay validation, the total bacterial burden in the original clinical samples was not determined in the present study. A direct head-to-head comparison with the *porA*-targeting real-time PCR assay reported by Hjelmevoll et al. was not performed; therefore, differences in assay format, reaction chemistry, template input, instrument platform, and validation design should be considered when interpreting analytical performance between the assays. Furthermore, the reaction conditions were selected through stepwise empirical screening rather than a full factorial optimization design. Potential interactions among reaction parameters, inter-run reproducibility under low-template conditions, and robustness across broader specimen matrices require further assessment. Within these boundaries, the present study provides laboratory-based qualitative detection data from directly extracted clinical specimens and reference strains, together with an exploratory Z017-calibrated molecular estimation framework evaluated in cultured WHO reference strains.

## 4. Materials and Methods

### 4.1. Study Design

This study was designed to establish and evaluate a defined *porA*-targeting TaqMan qPCR assay for the laboratory-based qualitative detection of *N. gonorrhoeae* and to evaluate its analytical and clinical applicability. The experimental workflow consisted of sequence design, in silico inclusivity and exclusivity assessment, qPCR condition selection through stepwise empirical screening, analytical specificity and sensitivity testing, validation using reference strains, validation using directly extracted clinical specimens, supportive retrospective qualitative agreement assessment, and standard-curve-based Z017-calibrated molecular estimation using cultured WHO reference strains.

### 4.2. Bacterial Strains and Reference Materials

*N. gonorrhoeae* reference strains were obtained from recognized culture collections, including ATCC, NCTC, and NCCP. The WHO gonococcal reference strains distributed through the NCTC were used for assay validation and Z017-calibrated molecular estimation experiments. A full list of the *N. gonorrhoeae* strains used in this study is provided in Table 5.

A titered *N. gonorrhoeae* Z017 preparation (ZeptoMetrix, Buffalo, NY, USA), supplied at 1.74 × 10^8^ CFU/mL in brain–heart infusion broth containing 15% glycerol, was used as the titered and quantified reference material for standard curve generation.

The non-gonococcal organisms used for analytical specificity testing included non-gonococcal *Neisseria* species and *C. trachomatis* serovar E. A full list of the non-target organisms used for analytical specificity testing is provided in Table 6. All bacterial stocks were stored at −80 °C until use.

### 4.3. Clinical Specimens

Clinical specimen-based validation was performed using *N. gonorrhoeae*-positive and -negative clinical specimens provided by Seoul Clinical Laboratory (Institutional Review Board [IRB] No. IRB-25-047). The validation set included 40 *N. gonorrhoeae*-positive urine specimens, 40 *N. gonorrhoeae*-positive vaginal swab specimens, 10 *N. gonorrhoeae*-negative urine specimens, and 10 *N. gonorrhoeae*-negative vaginal swab specimens.

The positive or negative status of each clinical specimen had been determined before acquisition using a careGene™ STD-12 detection kit (Wells Bio, Seoul, Republic of Korea). DNA was extracted from clinical specimens and used as a template for *porA*-targeting qPCR. In addition, a separate and independent retrospectively traceable urine specimen set comprising 70 specimens was used for supportive qualitative agreement assessment against routine Cobas 4800 CT/NG results. Specimens were included only when it could be linked one-to-one to the same primary specimen or to a documented matched aliquot using the study specimen identifier, specimen type, and collection or accession records. Specimens without an available Cobas result or without confirmed one-to-one linkage were excluded from the paired analysis. The Cobas comparator analysis was retrospective, descriptive, and intended to assess qualitative agreement within the traceably matched subset. It was not designed to establish formal diagnostic accuracy, clinical equivalence, or non-inferiority relative to the commercial comparator.

Clinical specimens were used only for qualitative detection validation and not for bacterial load estimation. Because the residual clinical specimens were not collected or processed for a quantitative analysis, they were not used to determine the total bacterial burden in the original clinical samples.

No dedicated experiment was performed to evaluate specimen-specific PCR inhibition, and the assay did not include an internal amplification control. Therefore, the clinical validation was not designed to characterize the extent of inhibition across urine and vaginal swab matrices. This study was approved by the Institutional Review Board of Dankook University Hospital (IRB No. 2024-11-012).

### 4.4. Culture of N. gonorrhoeae Reference Strains

**Figure 4:**
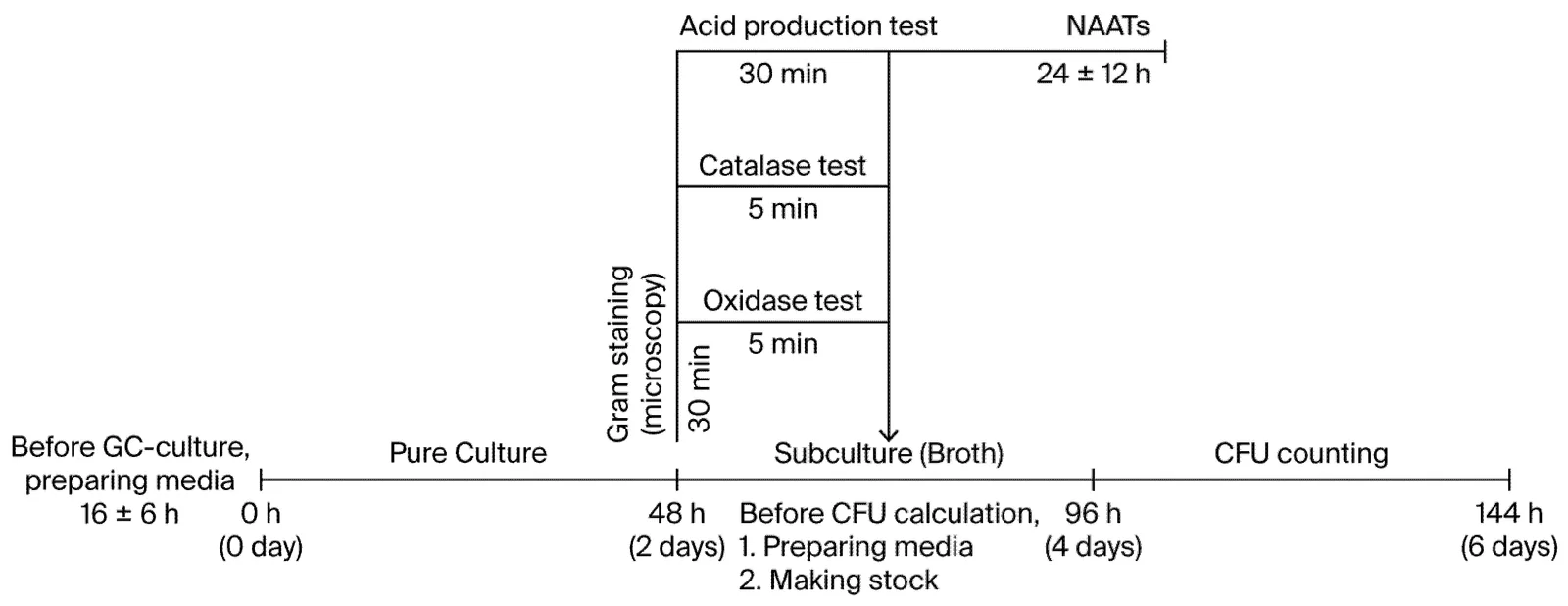
Schematic of the culture used for *Neisseria gonorrhoeae* reactivation and colony-forming unit counting.

Lyophilized *N. gonorrhoeae* reference strains were reconstituted according to the supplier’s instructions and cultured on chocolate agar with 1% Isovitale X. The plates were incubated at 37 °C in 5% CO_2_ for 48 h. Colonies showing typical gonococcal morphology were subcultured and used for downstream experiments (Figure 4).

To prepare the broth culture, confirmed colonies were inoculated in chocolate agar (GC) broth with 1% Isovitale X and incubated at 37 °C in 5% CO_2_. The broth cultures were used to determine viable cell counts and extract DNA. Bacterial stocks were prepared in 20% glycerol and stored at −80 °C.

### 4.5. Phenotypic and Molecular Identification of Cultured Strains

Cultured *N. gonorrhoeae* strains were confirmed using standard phenotypic and molecular identification methods. Phenotypic characterization included Gram staining, oxidase testing, catalase testing, and carbohydrate acid production testing. Molecular identification was performed using 16S rRNA gene amplification and sequencing.

Only confirmed *N. gonorrhoeae* cultures were used for the qPCR assay validation and Z017-calibrated qPCR-derived CFU/mL-equivalent estimation experiments.

### 4.6. Viable Count Determination by Culture

The viable counts of the cultured WHO reference strains were determined using serial dilutions and colony enumeration. The broth cultures were serially diluted 10-fold using sterile diluent, and 100 µL aliquots of each dilution were plated onto chocolate agar with 1% Isovitale X. The plates were incubated at 37 °C in 5% CO_2_ for 48 h. Plates containing 30–300 colonies were selected for enumeration. Each dilution was plated in triplicate, and CFU/mL values were calculated from the mean colony count. Plates showing contamination, excessive spreading, or uncountable growth were excluded from analysis. The same broth culture source was used for both CFU enumeration and DNA extraction for the Z017-calibrated qPCR-derived CFU/mL-equivalent estimation.

### 4.7. DNA Extraction from Bacterial Cultures and Clinical Specimens

Genomic DNA from the cultured bacterial strains was extracted from 200 µL of broth culture using a QIAamp DSP DNA Mini Kit (QIAGEN, Hilden, Germany) according to the manufacturer’s protocol. DNA was eluted in a final volume of 200 µL and stored at −20 °C until the qPCR analysis.

DNA was extracted from 200 µL of each urine and vaginal swab specimen using a QIAamp DSP DNA Mini Kit. The final elution volume was 200 µL for both specimen types. The extracted clinical DNA was used for the qualitative clinical specimen validation of the final *porA*-targeting qPCR assay conditions.

### 4.8. Primer and Probe Design and Empirical qPCR Condition Selection

The *porA* pseudogene was selected as the target region for *N. gonorrhoeae*-specific detection. *N. gonorrhoeae porA* sequences were retrieved from the NCBI and ATCC databases and aligned for primer and probe design. Candidate primer and probe sets were designed using Primer3 v4.1.0, and the corresponding sequence alignments were examined using BioEdit v7.2. The candidate oligonucleotide sequences, amplicon sizes, and preliminary screening results are provided in Supplementary Table S1 and Figure S1.

In silico inclusivity and exclusivity analyses of the selected primer–probe set were performed using NCBI BLASTn (https://blast.ncbi.nlm.nih.gov/Blast.cgi, accessed on 12 August 2026) and publicly available sequence records. Inclusivity was evaluated using 201 complete *N. gonorrhoeae* genome accessions retrieved from the NCBI genome database. Sequence identity was assessed separately for the forward primer, probe, and reverse primer, and the orientation and relative positions of the three oligonucleotide-binding sites were examined to determine whether the expected target amplicon could be formed. Accession-level results are provided in Supplementary Table S2.

Exclusivity was evaluated against 114 non-target organisms comprising bacterial, fungal, protozoal, and viral taxa selected as potential cross-reactive organisms. Individual oligonucleotide matches were assessed, and potential non-target amplification was evaluated according to the orientation and positions of the primer-binding sites, the presence of an intervening probe-binding site, and an expected amplicon length of less than 1 kb. Organism-level oligonucleotide match and amplicon assessment results are provided in Supplementary Table S3.

Two candidate primer and probe sets were designed and initially screened using DNA extracted from *N. gonorrhoeae* ATCC 19424. The final 157 bp probe set was selected because it showed more favorable amplification characteristics than the alternative 168 bp set under the tested conditions. For the selected probe set, qPCR conditions were evaluated through the sequential empirical screening of annealing/extension temperature and primer–probe concentration. The evaluated conditions are summarized in Table 1. The Ct value, fluorescence intensity, amplification curve morphology, and absence of amplification in the no-template control were considered during the selection of the final assay condition.

The final assay used the primer and probe set targeting a 157 bp region of the *porA* pseudogene: porA_F1: 5′-GAGGAAGCGGCTTGAATCTC-3′; porA_R1: 5′-ACAAAGTCGAAACCATGGGC-3′; porA_P1: 5′-FAM-TAATACAGTCCCGCGCATCA-BHQ1-3′. The assay condition selection experiments were performed in a stepwise manner and were not designed as a full factorial analysis of all the possible parameter interactions. The final conditions were therefore selected as the best-performing combination within the tested ranges.

The final qPCR reaction volume was 20 µL and consisted of 10 µL of 2× SsoAdvanced Universal Probes Supermix (Bio-Rad, Hercules, CA, USA), 1 µL of forward primer, 1 µL of reverse primer, 1 µL of FAM-labeled TaqMan probe, 6 µL of nuclease-free water, and 1 µL of template DNA. The final primer concentration was 500 nM for each forward and reverse primer, and the final probe concentration was 1000 nM. The final two-step qPCR cycling condition was as follows: initial denaturation at 95 °C for 3 min, followed by 40 cycles of denaturation at 95 °C for 5 s and annealing/extension at 60 °C for 30 s. Fluorescence was acquired at 60 °C. The total amplification runtime was 52 min and represents the on-instrument amplification time only.

All qPCR reactions were performed using the CFX Opus 96 Real-Time PCR System (Bio-Rad, Hercules, CA, USA) and analyzed using Bio-Rad CFX Maestro software (version 2.3.0; Bio-Rad, Hercules, CA, USA).

### 4.9. Assessment of Analytical Specificity and Sensitivity

Analytical specificity was evaluated using DNA extracted from non-gonococcal *Neisseria* species; *C. trachomatis* serovar E. *N. gonorrhoeae* ATCC 19424 DNA was used as the positive control, and nuclease-free water was used as the no-template control. Each target was analyzed in three technical replicates. Where amplification was observed, Ct values were summarized as the mean ± SD.

To assess the lowest observed detectable concentration in the original serial dilution experiment, 10-fold serial dilutions of *N. gonorrhoeae* ATCC 19424 DNA, ranging from 1.0 to 1 × 10^−8^ ng/µL, were analyzed. Each dilution was analyzed in three technical replicates. A fixed fluorescence threshold of 500 RFU was applied during data analysis. A dilution was classified as detected when its amplification curve crossed the threshold within the 40-cycle protocol and showed a typical sigmoidal amplification profile. The lowest concentration meeting these criteria was reported as the lowest observed detectable concentration under the tested conditions. Ct values obtained from replicate measurements were summarized as the mean ± SD.

### 4.10. Validation Using Reference Strains

The final qPCR assay conditions were evaluated using *N. gonorrhoeae* reference strains, including the WHO (NCTC), ATCC, and NCCP strains. The DNA extracted from each cultured strain was used as a template for the final two-step TaqMan qPCR assay.

A reaction was considered positive when the amplification curve crossed the 500-RFU fluorescence threshold within 40 cycles. Representative amplification curves from the reference strain validation experiment, including the no-template control curve, are provided in Supplementary Figure S2.

### 4.11. Validation Using Clinical Specimens

The clinical qualitative detection performance of the developed assay was evaluated using the DNA extracted directly from 100 clinical specimens: 40 *N. gonorrhoeae*-positive urine, 40 *N. gonorrhoeae*-positive vaginal, 10 negative urine, and 10 negative vaginal swab specimens.

Each clinical DNA extract was tested using the final *porA*-targeting two-step TaqMan qPCR assay. DNA was extracted from the original urine or vaginal swab specimens before the qPCR analysis. A fixed fluorescence threshold of 500 RFU was used to interpret the results. A specimen was classified as positive when the amplification curve crossed the 500-RFU threshold within 40 cycles and showed a typical sigmoidal amplification profile. Specimens without threshold crossing within 40 cycles or without a typical sigmoidal amplification profile were classified as negative.

The detection results were summarized separately for the positive urine specimens, positive vaginal swab specimens, negative urine controls, and negative vaginal swab controls. For the retrospectively traceable subset with routine Cobas 4800 CT/NG results, only specimens with confirmed one-to-one linkage to the same primary specimen or a documented matched aliquot were included in the paired comparator analysis. Overall, positive, and negative percent agreements were calculated with exact binomial 95% CIs, and Cohen’s kappa coefficient was calculated using the Cobas qualitative results as the comparator. Discordant results, when present, were summarized descriptively without additional resolution testing. No qPCR-derived molecular load estimates or clinical bacterial load cut-offs were generated from the clinical specimens.

### 4.12. Standard Curve Generation Using Titered Z017 Reference Material

For the exploratory qPCR-derived CFU/mL-equivalent estimation, titered *N. gonorrhoeae* Z017 reference material was used to generate a standard curve. Z017 was diluted to a working concentration of 1.00 × 10^7^ CFU/mL, and DNA was extracted from 200 µL of the diluted material. The extracted Z017 DNA was serially diluted 10-fold to generate standard curve points corresponding to 1.00 × 10^7^ to 1.00 × 10^2^ CFU/mL-equivalent concentrations. Each standard dilution was analyzed in triplicate using the final two-step qPCR assay.

Ct values were plotted against log10-transformed CFU/mL-equivalent concentrations using Bio-Rad CFX Maestro software. For each standard concentration, the mean Ct value and SD were calculated from three technical replicates. The standard curve was computed using the mean Ct values, and SD error bars were generated (Figure 3A). Individual Ct values, mean Ct values, SDs, and coefficients of variation are provided in Supplementary Table S6. The standard curve was evaluated based on the coefficient of determination (R^2^), slope, intercept, and amplification efficiency.

To assess the effect of the order of extraction and dilution on the Z017 preparation workflow, Ct values were compared between two procedures: DNA extraction, followed by the serial dilution of the extracted DNA, and the serial dilution of the titered Z017 material, followed by DNA extraction from each dilution. The mean Ct difference between the two workflows was calculated.

### 4.13. qPCR-Based Molecular Estimation of the Cultured Reference Strains

The final *porA*-targeting probe set and Z017-derived standard curve were used for the qPCR-derived CFU/mL-equivalent molecular estimation of the cultured *N. gonorrhoeae* reference strains. The analysis was performed using cultured WHO reference strains (Table 7).

For each strain, viable count determination via CFU enumeration and qPCR-based CFU/mL-equivalent molecular estimation were performed using aliquots derived from the same broth culture. The DNA extracted from each broth culture was analyzed using the final qPCR assay conditions. For each cultured WHO reference strain, the mean Ct value obtained from triplicate qPCR measurements was entered into the regression equation generated from the Z017 standard curve to calculate the corresponding qPCR-derived CFU/mL-equivalent estimate.

All qPCR-based molecular estimation experiments were performed in triplicate. For each strain, the mean qPCR-derived CFU/mL-equivalent estimate, SD, and coefficient of variation were calculated. For strain-level comparisons, the culture/qPCR ratio was calculated as the culture-derived CFU/mL value divided by the qPCR-derived CFU/mL-equivalent estimate.

Because qPCR detects target DNA, whereas CFU enumeration measures viable and culturable bacteria, qPCR-derived values were reported as Z017-calibrated, standard-curve-based CFU/mL-equivalent molecular estimates rather than as direct measures of viable CFUs.

### 4.14. Statistical Analysis

Each clinical specimen was tested three times. Standard curve generation was performed in triplicate, and qPCR-based CFU/mL-equivalent molecular estimation experiments using cultured WHO reference strains were also performed in triplicate.

Ct values from triplicate experiments are presented as the mean ± SD. The coefficient of variation was calculated as follows:

CV (%) = (SD/mean) × 100

Standard curve analyses were performed using Bio-Rad CFX Maestro software. The mean values and SDs were calculated using Microsoft Excel v16.106. For the qPCR-based molecular estimation experiments, Ct values were converted to qPCR-derived CFU/mL-equivalent estimates by interpolation from a Z017-derived standard curve.

For descriptive comparisons with culture-derived CFU/mL values, paired qPCR-derived CFU/mL-equivalent estimates and culture-derived CFU/mL values from the 12 cultured WHO reference strains were log10-transformed. Pearson’s correlation coefficient was calculated, and the mean log10 difference was calculated as log10(qPCR-derived CFU/mL-equivalent estimate) − log10(culture-derived CFU/mL). The overall culture/qPCR fold difference was calculated as the geometric mean of the strain-level culture/qPCR ratios, equivalent to 10 raised to the mean value of log10(culture-derived CFU/mL) − log10(qPCR-derived CFU/mL-equivalent estimate). These analyses were used to describe the association and average quantitative difference between the two measurements and were not intended to establish method interchangeability.

The clinical specimen validation results are summarized as the number and percentage of positive or negative results in each specimen group. For the retrospective Cobas 4800 CT/NG comparison, positive, negative, and overall percent agreements were calculated with exact binomial 95% CIs. Cohen’s kappa coefficient was calculated to describe the qualitative agreement between the developed assay and Cobas 4800 CT/NG testing.

## 5. Conclusions

In this study, we evaluated a *porA*-targeting two-step TaqMan qPCR workflow as a laboratory-based method for the qualitative detection of *N. gonorrhoeae*. In silico analysis supported the sequence coverage and target discrimination of the selected probe set across 201 *N. gonorrhoeae* genome accessions and 114 non-target organisms. The assay was assessed using 23 *N. gonorrhoeae* reference strains and DNA extracted from primary urine and vaginal swab specimens. Under the tested conditions, the assay detected all evaluated reference strains, classified all 80 *N. gonorrhoeae*-positive clinical specimens as positive, and showed no amplification in the 20 negative clinical specimens. The 52 min duration represents the on-instrument qPCR amplification runtime only and does not indicate the complete sample-to-answer time.

A Z017-derived standard curve was additionally applied to cultured WHO reference strains to generate qPCR-derived CFU/mL-equivalent molecular estimates. The triplicate measurements showed reproducible Ct values; however, all qPCR-derived estimates were lower than the paired culture-derived CFU/mL values. The geometric mean culture/qPCR ratio was 15.35:1, and the log10-transformed paired values were not significantly correlated (Pearson’s r = −0.456, *p* = 0.136). These findings characterize the method-dependent relationship between Z017-calibrated molecular estimates and culture-derived viable counts and support their interpretation as complementary molecular estimates rather than direct equivalents of viable CFUs.

Overall, this study provides an assay design and validation-oriented laboratory framework that combines in silico sequence assessment, *porA*-targeting qualitative detection in clinical specimens, and exploratory Z017-calibrated molecular estimation in cultured WHO reference strains. The assay is intended to complement established commercial NAATs and culture-based antimicrobial susceptibility testing.

## Figures and Tables

**Table 2:** Validation of the final assay conditions using *Neisseria gonorrhoeae* reference strains.

Strain	WHO Strain	Ct Value	Result
ATCC 19424		21.26	Positive
ATCC 31426		21.20	Positive
ATCC 49226		21.21	Positive
ATCC 49981		21.14	Positive
NCTC 13477	F	21.44	Positive
NCTC 13478	G	21.50	Positive
NCTC 13479	K	21.14	Positive
NCTC 13480	L	23.68	Positive
NCTC 13481	M	21.36	Positive
NCTC 13482	N	22.80	Positive
NCTC 13483	O	21.49	Positive
NCTC 13484	P	21.08	Positive
NCTC 14208	Q	21.73	Positive
NCTC 15801	H	21.57	Positive
NCTC 15802	R	22.24	Positive
NCTC 15803	S2	22.95	Positive
NCCP 11343		17.21	Positive
NCCP 11351		18.54	Positive
NCCP 11363		18.84	Positive
NCCP 11395		18.11	Positive
NCCP 11937		18.68	Positive
NCCP 16057		18.32	Positive
NCCP 16058		18.62	Positive
No-template control		None	Negative

Each strain was tested in three technical replicates. The Ct values shown represent the means of the three technical replicates, and representative amplification curves are provided in Supplementary Figure S2.

**Table 3:** Paired qualitative agreement between the developed *porA*-targeting TaqMan qPCR assay and Cobas 4800 CT/NG testing in a separate retrospective urine specimen subset.

Cobas 4800 CT/NG Result	Developed qPCR Positive	Developed qPCR Negative	Total
*N. gonorrhoeae*-positive	30	0	30
Negative	0	40	40
Total	30	40	70

The retrospective urine specimen subset was independent of the original 100-specimen clinical validation set. Positive percent agreement was 100% (30/30; 95% confidence interval [CI], 88.4–100%), negative percent agreement was 100% (40/40; 95% CI, 91.2–100%), and overall percent agreement was 100% (70/70; 95% CI, 94.9–100%). Cohen’s kappa coefficient was 1.00.

**Table 4:** qPCR-derived CFU/mL-equivalent estimates and culture-derived CFU counts of cultured WHO reference strains.

Strain	WHO Strain	Mean Ct ± Standard Deviation	qPCR-Derived Estimate (CFU/mL-Equivalent)		Culture-Derived Count (CFU/mL)	Culture/qPCR Ratio	Log10 Difference: qPCR − Culture
NCTC 13477	F	19.81 ± 0.01	2.17 × 10^6^		8.26 × 10^6^	3.80	−0.580
NCTC 13478	G	20.02 ± 0.13	1.89 × 10^6^		4.21 × 10^7^	22.29	−1.348
NCTC 13479	K	20.62 ± 0.04	1.27 × 10^6^		2.74 × 10^7^	21.64	−1.335
NCTC 13480	L	19.84 ± 0.09	2.13 × 10^6^		8.11 × 10^6^	3.80	−0.580
NCTC 13481	M	22.65 ± 0.73	3.28 × 10^5^		1.22 × 10^8^	370.98	−2.569
NCTC 13482	N	19.70 ± 0.01	2.34 × 10^6^		5.28 × 10^7^	22.59	−1.354
NCTC 13483	O	20.53 ± 0.22	1.35 × 10^6^		2.94 × 10^7^	21.82	−1.339
NCTC 13484	P	19.98 ± 0.21	1.94 × 10^6^		4.37 × 10^7^	22.51	−1.352
NCTC 14208	Q	19.24 ± 0.09	3.18 × 10^6^		1.14 × 10^7^	3.59	−0.555
NCTC 15801	H	20.80 ± 0.19	1.13 × 10^6^		2.95 × 10^7^	26.26	−1.419
NCTC 15802	R	21.01 ± 0.11	9.78 × 10^5^		8.49 × 10^6^	8.68	−0.938
NCTC 15803	S2	20.67 ± 0.41	1.23 × 10^6^		8.91 × 10^6^	7.27	−0.861
Geometric mean ratio				15.35:1			

The culture/qPCR ratio was calculated as culture-derived CFU/mL divided by the qPCR-derived CFU/mL-equivalent estimate. The geometric mean ratio was calculated from the strain-specific culture/qPCR ratios. The log10 difference was calculated as log10(qPCR-derived CFU/mL-equivalent estimate) − log10(culture-derived CFU/mL).

**Table 5:** *Neisseria gonorrhoeae* reference strains used for assay evaluation.

Bacteria	Culture Collection	Strain	The WHO Strain	Accession Number
*Neisseria gonorrhoeae*	American Type Culture Collection (ATCC)	ATCC 19424	N/A	N/A
		ATCC 31426	N/A	N/A
		ATCC 49226	N/A	N/A
		ATCC 49981	N/A	N/A
	National Collection of Type Cultures (NCTC)	NCTC 13477	F	CP145052.1
		NCTC 13478	G	CP145077.1
		NCTC 13479	K	CP145048.1
		NCTC 13480	L	CP145045.1
		NCTC 13481	M	CP145041.1
		NCTC 13482	N	CP145067.1
		NCTC 13483	O	CP145037.1
		NCTC 13484	P	CP145035.1
		NCTC 14208	Q	CP145032.1
		NCTC 15801	H	CP145050.1
		NCTC 15802	R	CP145028.1
		NCTC 15803	S2	CP145026.1
	National Culture Collection for Pathogens (NCCP)	NCCP 11343	N/A	N/A
		NCCP 11351	N/A	N/A
		NCCP 11363	N/A	N/A
		NCCP 11395	N/A	N/A
		NCCP 11937	N/A	N/A
		NCCP 16057	N/A	N/A
		NCCP 16058	N/A	N/A

N/A: Genome sequences were collected from the ATCC Genome Browser and used for in silico testing.

**Table 6:** Non-gonococcal organisms used for analytical specificity testing.

Bacteria	Culture Collection	Strain	Serovar
*Neisseria oralis*	Korean Collection for Type Cultures (KCTC)	KCTC 15271	
*N. dentiae*		KCTC 23249T	
*N. shayeganii*		KCTC 25501T	
*N. flavescens*		KCTC 52592T	
*N.* sp.		KCTC 5520	
*N. yagbaofengii*		KCTC 82336T	
*N. meningitidis*	Korean Culture Center of Microorganisms (KCCM)	KCCM 41562	
*N.* sp. *KCOM 1440*		KCCM 42783	
*N. flava MA1-1*		KCCM 43414	
*N. subflava MA3-1*	ATCC	ATCC 13100	
*Chlamydia trachomatis*	NCCP	NCCP 17628	E

**Table 7:** Cultured WHO reference strains for Z017-calibrated qPCR-derived CFU/mL-equivalent molecular estimation, Gram staining, 16S rRNA sequencing, oxidase testing, catalase testing, acid production testing, and colony-forming unit counting.

Strain	WHO Strain	Gram Stain	16S rRNA	Oxidase	Catalase	Acid Production				Concentration (CFU/mL)
						GLU ^†^	MAL ^‡^	SUC ^††^	LAC ^‡‡^	
NCTC 13477	F	GND	NG+	+	+	+	−	−	−	1.09 × 10^8^
NCTC 13478	G	GND	NG+	+	+	+	−	−	−	8.90 × 10^7^
NCTC 13479	K	GND	NG+	+	+	+	−	−	−	1.39 × 10^8^
NCTC 13480	L	GND	NG+	+	+	+	−	−	−	7.26 × 10^7^
NCTC 13481	M	GND	NG+	+	+	+	−	−	−	4.83 × 10^6^
NCTC 13482	N	GND	NG+	+	+	+	−	−	−	1.01 × 10^8^
NCTC 13483	O	GND	NG+	+	+	+	−	−	−	8.80 × 10^7^
NCTC 13484	P	GND	NG+	+	+	+	−	−	−	3.46 × 10^8^
NCTC 14208	Q	GND	NG+	+	+	+	−	−	−	2.10 × 10^7^
NCTC 15801	H	GND	NG+	+	+	+	−	−	−	8.97 × 10^7^
NCTC 15802	R	GND	NG+	+	+	+	−	−	−	3.87 × 10^7^
NCTC 15803	S2	GND	NG+	+	+	+	−	−	−	3.43 × 10^7^

GND: Gram-negative Diplococci; ^†^: glucose, ^‡^: maltose, ^††^: sucrose, ^‡‡^: lactose; +: positive result on a biochemical test, −: negative result on a biochemical test.

## Data Availability

All data supporting the findings of this study are included in the article and its Supplementary Materials. *N. gonorrhoeae* and reference strain genomes were obtained from GenBank, and the GenBank accession numbers for each sequence are provided in the manuscript. The original contributions of this study are included in the article and Supplementary Materials. Further inquiries may be directed to the corresponding authors.

## Supplementary Materials

The following supporting information can be downloaded at: https://www.mdpi.com/article/10.3390/ijms27167335/s1.
