# Supplementary material for: Development and Laboratory Evaluation of a porA-Targeting TaqMan qPCR Assay for Neisseria gonorrhoeae: Clinical Specimen Validation and Molecular Estimation Using Cultured Reference Strains
Source: Int J Mol Sci. 2026 Aug 17;27(16):7335. doi: 10.3390/ijms27167335 (PMC13512994; doi:10.3390/ijms27167335)
Supplement: Supplementary file 1 [file ijms-27-07335-s001.zip › Revision3_ijms-4370247_Supplementary data.pdf]

Supplementary Data

Supplementary Table S1. Candidate *porA* probe sets evaluated for preliminary *Neisseria gonorrhoeae* screening using qPCR.

Set	Target	Name	Sequence (5′→3′)	GC (%)	Tm (°C)	Product size (bp)	Preliminary Ct	Maximum Fluorescence (RFU)
Set 1	*porA* pseudogene	porA_F1	GAGGAAGCGGCTTGAATCTC	55.0	55.8	157	21.54	12,000
		porA_R1	ACAAAGTCGAAACCATGGGC	50.0	56.0			
		porA_P1	FAM-TAATACAGTCCCGCGCATCA-BHQ1	50.0	56.3			
Set 2	*porA* pseudogene	porA_F2	GTAATACAGTCCCGCGCATC	55.0	55.8	168	22.75	4,500
		porA_R2	TGCCGGTATTTCGTTTCAGC	50.0	50.0			
		porA_P2	FAM-ACGATTTTTCCAAGCGCACT-BHQ1	45.0	55.7			

Ct values and maximum fluorescence signals were obtained during preliminary screening using DNA extracted from *N. gonorrhoeae* ATCC 19424. The annealing/extension temperature was found to be 60°C for Set 1 and 58°C for Set 2. No amplification was observed in the no-template control.

Supplementary Table S2. Accession-level in-silico inclusivity analysis of the selected *porA*-targeting probe set against 201 *Neisseria gonorrhoeae* genomes.

No.	Accession No.	Forward primer identity (%)	Probe identity (%)	Reverse primer identity (%)	Compatible arrangement for expected amplicon
1	AE004969.1	100	100	100	Yes
2	AP018377.1	100	100	100	Yes
3	AP018381.1	100	100	100	Yes
4	AP018385.1	100	100	100	Yes
5	AP019853.2	100	100	100	Yes
6	AP023067.1	100	100	100	Yes
7	AP023069.1	100	100	100	Yes
8	AP023071.1	100	100	100	Yes
9	AP023073.1	100	100	100	Yes
10	AP023075.1	100	100	100	Yes
11	AP024155.1	100	100	100	Yes
12	CP001050.1	100	100	100	Yes
13	CP003909.1	100	100	100	Yes
14	CP003974.1	100	100	100	Yes
15	CP012026.1	100	100	100	Yes
16	CP012027.1	100	100	100	Yes
17	CP012028.1	100	100	100	Yes
18	CP016015.1	100	100	100	Yes
19	CP016016.1	100	100	100	Yes
20	CP016017.1	100	100	100	Yes
21	CP019466.1	100	100	100	Yes
22	CP019467.1	100	100	100	Yes
23	CP020415.2	100	100	100	Yes
24	CP020418.2	100	100	100	Yes
25	CP020419.2	100	100	100	Yes

26	CP032398.2	100	100	100	Yes
27	CP032429.2	100	100	100	Yes
28	CP034016.1	100	100	100	Yes
29	CP034018.1	100	100	100	Yes
30	CP034020.1	100	100	100	Yes
31	CP034022.1	100	100	100	Yes
32	CP034024.1	100	100	100	Yes
33	CP034026.1	100	100	100	Yes
34	CP034028.1	100	100	100	Yes
35	CP034030.1	100	100	100	Yes
36	CP034032.1	100	100	100	Yes
37	CP041585.1	100	100	100	Yes
38	CP041586.1	100	100	100	Yes
39	CP043812.1	100	100	100	Yes
40	CP043816.1	100	100	100	Yes
41	CP043871.1	100	100	100	Yes
42	CP045707.1	100	100	100	Yes
43	CP045832.1	100	100	100	Yes
44	CP048246.1	100	100	100	Yes
45	CP048250.1	95	100	100	Yes
46	CP048254.1	100	100	100	Yes
47	CP048893.1	100	100	100	Yes
48	CP048896.1	100	100	100	Yes
49	CP048899.1	100	100	100	Yes
50	CP048902.1	100	100	100	Yes
51	CP048905.1	100	100	100	Yes
52	CP048908.1	100	100	100	Yes
53	CP048911.1	100	100	100	Yes
54	CP048929.1	100	100	100	Yes

55	CP050505.1	100	100	100	Yes
56	CP050930.1	100	100	100	Yes
57	CP068761.1	100	100	100	Yes
58	CP078113.1	100	100	100	Yes
59	CP078114.1	100	100	100	Yes
60	CP078115.1	100	100	100	Yes
61	CP078116.1	100	100	100	Yes
62	CP078117.1	100	100	100	Yes
63	CP078118.1	100	100	100	Yes
64	CP078119.1	100	100	100	Yes
65	CP097846.1	100	100	100	Yes
66	CP098458.1	100	100	100	Yes
67	CP098460.1	100	100	100	Yes
68	CP098462.1	100	100	100	Yes
69	CP098464.2	100	100	100	Yes
70	CP098466.2	100	100	100	Yes
71	CP098468.2	100	100	100	Yes
72	CP098470.2	100	100	100	Yes
73	CP098472.2	100	100	100	Yes
74	CP098474.1	100	100	100	Yes
75	CP098503.2	100	100	100	Yes
76	CP098505.2	100	100	100	Yes
77	CP098532.2	100	100	100	Yes
78	CP098534.2	100	100	100	Yes
79	CP098536.1	100	100	100	Yes
80	CP098538.2	100	100	100	Yes
81	CP098542.2	100	100	100	Yes
82	CP098544.1	100	100	100	Yes
83	CP098546.2	100	100	100	Yes

84	CP098548.2	100	100	100	Yes
85	CP104546.2	100	100	100	Yes
86	CP104548.2	100	100	100	Yes
87	CP106749.1	100	100	100	Yes
88	CP106765.1	100	100	100	Yes
89	CP106767.1	100	100	100	Yes
90	CP106769.1	100	100	100	Yes
91	CP106771.1	100	100	100	Yes
92	CP106773.1	100	100	100	Yes
93	CP106775.1	100	100	100	Yes
94	CP106777.1	100	100	100	Yes
95	CP106780.1	100	100	100	Yes
96	CP106782.1	100	100	100	Yes
97	CP106789.1	100	100	100	Yes
98	CP106801.1	100	100	100	Yes
99	CP106803.1	100	100	100	Yes
100	CP106805.1	100	100	100	Yes
101	CP106807.1	100	100	100	Yes
102	CP106809.1	100	100	100	Yes
103	CP106811.1	100	100	100	Yes
104	CP106813.1	100	100	100	Yes
105	CP106815.1	100	100	100	Yes
106	CP106817.1	100	100	100	Yes
107	CP106819.1	100	100	100	Yes
108	CP106821.1	100	100	100	Yes
109	CP106824.1	100	100	100	Yes
110	CP106826.1	100	100	100	Yes
111	CP106918.1	100	100	100	Yes
112	CP106921.1	100	100	100	Yes

113	CP107223.1	100	100	100	Yes
114	CP107264.1	100	100	100	Yes
115	CP107266.1	100	100	100	Yes
116	CP107270.1	100	100	100	Yes
117	CP107271.1	100	100	100	Yes
118	CP107272.1	100	100	100	Yes
119	CP107274.1	100	100	100	Yes
120	CP115654.1	100	100	100	Yes
121	CP115904.1	100	100	100	Yes
122	CP116342.1	100	100	100	Yes
123	CP119140.1	100	100	100	Yes
124	CP119142.1	100	100	100	Yes
125	CP130889.1	100	100	100	Yes
126	CP130890.1	100	100	100	Yes
127	CP130891.1	100	100	100	Yes
128	CP130892.1	100	100	100	Yes
129	CP130893.1	100	100	100	Yes
130	CP130894.1	100	100	100	Yes
131	CP130895.1	100	100	100	Yes
132	CP130896.1	100	100	100	Yes
133	CP130897.1	100	100	100	Yes
134	CP130898.1	100	100	100	Yes
135	CP130899.1	100	100	100	Yes
136	CP131616.1	100	100	100	Yes
137	CP131620.1	100	100	100	Yes
138	CP131622.1	100	100	100	Yes
139	CP131626.1	100	100	100	Yes
140	CP131630.1	100	100	100	Yes
141	CP131632.1	100	100	100	Yes

142	CP131636.1	100	100	100	Yes
143	CP131638.1	100	100	100	Yes
144	CP131641.1	100	100	100	Yes
145	CP131645.1	100	100	100	Yes
146	CP138339.1	100	100	100	Yes
147	CP138342.1	100	100	100	Yes
148	CP145015.1	100	100	100	Yes
149	CP145017.1	100	100	100	Yes
150	CP145019.1	100	100	100	Yes
151	CP145021.1	100	100	100	Yes
152	CP145024.1	100	100	100	Yes
153	CP145026.1	100	100	100	Yes
154	CP145028.1	100	100	100	Yes
155	CP145032.1	100	100	100	Yes
156	CP145035.1	100	100	100	Yes
157	CP145037.1	100	100	100	Yes
158	CP145041.1	100	100	100	Yes
159	CP145045.1	100	100	100	Yes
160	CP145048.1	100	100	100	Yes
161	CP145050.1	100	100	100	Yes
162	CP145052.1	100	100	100	Yes
163	CP145053.1	100	100	100	Yes
164	CP145057.1	100	100	100	Yes
165	CP145059.1	100	100	100	Yes
166	CP145062.1	100	100	100	Yes
167	CP145064.1	100	100	100	Yes
168	CP145067.1	100	100	100	Yes
169	CP145071.1	100	100	100	Yes
170	CP145074.1	100	100	100	Yes

171	CP145077.1	100	100	100	Yes
172	CP145080.1	100	100	100	Yes
173	CP145083.1	100	100	100	Yes
174	CP145086.1	100	100	100	Yes
175	CP145088.1	100	100	100	Yes
176	CP145090.1	100	100	100	Yes
177	CP154352.1	100	100	100	Yes
178	CP173226.1	100	100	100	Yes
179	LR134358.1	100	100	100	Yes
180	LT591897.1	100	100	100	Yes
181	LT591898.1	100	100	100	Yes
182	LT591901.1	100	100	100	Yes
183	LT591904.1	100	100	100	Yes
184	LT591908.1	100	100	100	Yes
185	LT591910.1	100	100	100	Yes
186	LT592146.1	100	100	100	Yes
187	LT592150.1	100	100	100	Yes
188	LT592153.1	100	100	100	Yes
189	LT592155.1	100	100	100	Yes
190	LT592157.1	100	100	100	Yes
191	LT592159.1	100	100	100	Yes
192	LT592161.1	100	100	100	Yes
193	LT592163.1	100	100	100	Yes
194	LT906437.1	100	100	100	Yes
195	LT906440.1	100	100	100	Yes
196	LT906472.1	100	100	100	Yes
197	OZ004876.1	100	100	100	Yes
198	OZ021673.1	100	100	100	Yes
199	OZ021676.1	100	100	100	Yes

200	OZ197910.1	100	100	100	Yes
201	OZ197914.1	100	100	100	Yes

Sequence identity was assessed separately for the forward primer, probe, and reverse primer. All 201 accessions retained a compatible forward primer–probe–reverse primer arrangement for the expected amplicon. CP048250.1 showed 95% forward-primer identity; all other oligonucleotide identities were 100%. The analysis used NCBI sequence records available as of 10 February 2025.

Supplementary Table S3. Organism-level in-silico exclusivity analysis of the selected *porA*-targeting probe set against 114 non-target organisms.

No.	Organism	Taxonomy ID	Forward primer match	Probe match	Reverse primer match	Compatible expected amplicon
1	*Candida albicans*	5476	**< 85.00%**<br>CP025151.1<br>1090396 - 1090415	**< 80.00%**<br>CP025152.1<br>595440 - 595459	**< 75.00%**<br>AL033501.1<br>16856 - 16875	No compatible expected amplicon
2	*Corynebacterium diphtheriae*	1717	**< 85.00%**<br>CP085994.1<br>1526159 - 1526178	**< 85.00%**<br>BX248358.1<br>1067 - 1086	**< 75.00%**<br>BX248355.1<br>262833 - 262852	No compatible expected amplicon
3	*Enterococcus faecalis*	1351	**< 90.00%**<br>AP017623.1<br>2673132 - 2673151	**< 80.00%**<br>MK791686.1<br>1092 - 1111	**< 80.00%**<br>AB374546.1<br>47001 - 47020	No compatible expected amplicon
4	*Enterococcus faecium*	1352	**< 85.00%**<br>AP026566.1<br>2131369 - 2131388	**< 90.00%**<br>MG002173.1<br>1037 - 1056	**< 75.00%**<br>CP025685.1<br>1202381 - 1202400	No compatible expected amplicon
5	*Escherichia coli*	562	**< 85.00%**<br>AB005050.1<br>205 - 224	**< 90.00%**<br>AP018395.1<br>1628025 - 1628044	**< 75.00%**<br>CP013193.1<br>1175 - 1194	No compatible expected amplicon
6	*Fusobacterium necrophorum*	859	**< 85.00%**<br>CP033837.1<br>2294834 - 2294853	**< 85.00%**<br>DQ440554.1<br>1161 - 1180	**< 75.00%**<br>CP033837.1<br>88762 - 88781	No compatible expected amplicon
7	*Fusobacterium nucleatum*	851	**< 80.00%**<br>—<br>—	**< 80.00%**<br>—<br>—	**< 75.00%**<br>—<br>—	No compatible expected amplicon
8	*Haemophilus influenzae*	727	**< 85.00%**<br>AP018764.1<br>1448502 - 1448521	**< 80.00%**<br>AP018776.1<br>820109 - 820128	**< 80.00%**<br>AP018764.1<br>998193 - 998212	No compatible expected amplicon
9	*Mycoplasmoides pneumoniae FH*	722438	**< 80.00%**<br>CP002077.1<br>211290 - 211309	**< 75.00%**<br>AF132740.1<br>1143 - 1162	**< 85.00%**<br>CP002077.1<br>195898 - 195917	No compatible expected amplicon
10	*Porphyromonas gingivalis*	837	**< 85.00%**<br>CP007756.1<br>1324768 - 1324787	**< 80.00%**<br>CP024598.1<br>609534 - 609553	**< 80.00%**<br>CP007756.1<br>1676327 - 1676346	No compatible expected amplicon
11	*Pseudomonas aeruginosa*	287	**< 85.00%**<br>CP077981.1<br>1292366 - 1292385	**< 85.00%**<br>CP030910.1<br>3240574 - 3240593	**< 80.00%**<br>CP007399.1<br>648217 - 648236	No compatible expected amplicon
12	*Pseudomonas paraeruginosa*	2994495	**< 80.00%**<br>—<br>—	**< 80.00%**<br>—<br>—	**< 75.00%**<br>—<br>—	No compatible expected amplicon

No.	Organism	Taxonomy ID	Forward primer match	Probe match	Reverse primer match	Compatible expected amplicon
13	*Salmonella enterica*	28901	**< 85.00%** CP028169.1 2192267 - 2192286	**< 85.00%** CP053577.1 79210 - 79229	**< 85.00%** CP042438.1 4393548 - 4393567	No compatible expected amplicon
14	*Staphylococcus epidermidis*	1282	**< 85.00%** CP013943.1 1469530 - 1469549	**< 85.00%** LR735429.1 2241572 - 2241591	**< 75.00%** AF269854.1 1920 - 1939	No compatible expected amplicon
15	*Staphylococcus haemolyticus*	1283	**< 85.00%** AM399081.1 70 - 89	**< 85.00%** CP035291.1 2406134 - 2406153	**< 80.00%** CP033814.1 414764 - 414783	No compatible expected amplicon
16	*Staphylococcus hominis*	1290	**< 80.00%** — —	**< 80.00%** — —	**< 80.00%** — —	No compatible expected amplicon
17	*Streptococcus agalactiae*	1311	**< 85.00%** OX461113.1 436485 - 436504	**< 75.00%** AB002480.1 1124 - 1143	**< 75.00%** CP138364.1 430509 - 430528	No compatible expected amplicon
18	*Streptococcus dysgalactiae*	1334	**< 80.00%** — —	**< 75.00%** — —	**< 80.00%** — —	No compatible expected amplicon
19	*Streptococcus mutans*	1309	**< 80.00%** — —	**< 80.00%** — —	**< 80.00%** — —	No compatible expected amplicon
20	*Streptococcus sanguinis*	1305	**< 80.00%** — —	**< 80.00%** — —	**< 80.00%** — —	No compatible expected amplicon
21	*Veillonella parvula*	29466	**< 85.00%** CP019721.1 23384 - 23403	**< 80.00%** CP019721.1 874687 - 874706	**< 85.00%** CP138632.1 1821194 - 1821213	No compatible expected amplicon
22	*Burkholderia multivorans*	87883	**< 80.00%** CP008729.1 51248 - 51267	**< 85.00%** CP090733.1 847613 - 847632	**< 85.00%** CP013431.1 981206 - 981225	No compatible expected amplicon
23	*Citrobacter freundii*	546	**< 85.00%** AP026940.1 1657054 - 1657073	**< 90.00%** CP047307.1 2554823 - 2554842	**< 80.00%** AP014939.1 144953 - 144972	No compatible expected amplicon
24	*Paraclostridium sordellii*	1505	**< 85.00%** CP014150.1 184133 - 184152	**< 75.00%** AB448941.1 990 - 1009	**< 80.00%** CP014150.1 81126 - 81145	No compatible expected amplicon
25	*Enterobacter cloacae*	550	**< 85.00%** AP022126.1 3671081 - 3671100	**< 80.00%** CP009756.1 4229036 - 4229055	**< 80.00%** AP022126.1 70509 - 70528	No compatible expected amplicon
26	*Giardia intestinalis*	5741	**< 85.71%** CP110916.1 855766 - 855786	**< 85.00%** CP110920.1 1759691 - 1759710	**< 80.00%** CP110916.1 640273 - 640292	No compatible expected amplicon
27	*Lactobacillus acidophilus*	1579	**< 80.00%** — —	**< 80.00%** — —	**< 75.00%** — —	No compatible expected amplicon

No.	Organism	Taxonomy ID	Forward primer match	Probe match	Reverse primer match	Compatible expected amplicon
28	*Proteus mirabilis*	584	**< 85.00%** AP026827.1 1055781 - 1055800	**< 90.00%** CP026062.1 3673333 - 3673352	**< 80.00%** CP023273.1 3404 - 3423	No compatible expected amplicon
29	*Serratia marcescens*	615	**< 85.00%** AP028482.1 11892 - 11911	**< 90.00%** AP028531.1 3196057 - 3196076	**< 80.00%** AP024916.1 4268747 - 4268766	No compatible expected amplicon
30	*Staphylococcus aureus*	1280	**< 85.00%** CP033506.1 1623873 - 1623892	**< 85.00%** MG546551.1 1169 - 1188	**< 75.00%** CP009828.1 810043 - 810062	No compatible expected amplicon
31	*Trichomonas vaginalis*	5722	**< 75.00%** — —	**< 80.00%** — —	**< 70.00%** — —	No compatible expected amplicon
32	*Ureaplasma parvum*	134821	**< 75.00%** — —	**< 75.00%** — —	**< 70.00%** — —	No compatible expected amplicon
33	*Vibrio parahaemolyticus*	670	**< 90.00%** CP009765.1 41278 - 41297	**< 80.00%** CP009765.1 347298 - 347317	**< 80.00%** AB455531.1 32952 - 32971	No compatible expected amplicon
34	*Human adenovirus 40*	28284	**< 70.00%** — —	**< 70.00%** — —	**< 65.00%** — —	No compatible expected amplicon
35	*Cytomegalovirus*	10358	**< 75.00%** — —	**< 75.00%** — —	**< 80.00%** — —	No compatible expected amplicon
36	*Human alphaherpesvirus 1*	10298	**< 75.00%** — —	**< 70.00%** — —	**< 55.00%** — —	No compatible expected amplicon
37	*Human alphaherpesvirus 2*	10310	**< 70.00%** — —	**< 70.00%** — —	**< 75.00%** — —	No compatible expected amplicon
38	*Japanese encephalitis virus*	11072	**< 80.00%** — —	**< 70.00%** — —	**< 75.00%** — —	No compatible expected amplicon
39	*Bacillus subtilis*	1423	**< 90.00%** CP121705.1 691024 - 691043	**< 85.00%** AB677951.1 1212 - 1231	**< 75.00%** AP024621.1 2383003 - 2383022	No compatible expected amplicon
40	*Bifidobacterium adolescentis*	1680	**< 85.00%** CP007443.1 1414095 - 1414114	**< 80.00%** CP023005.1 1214485 - 1214504	**< 80.00%** CP007443.1 548351 - 548370	No compatible expected amplicon
41	*Campylobacter coli*	195	**< 80.00%** — —	**< 80.00%** — —	**< 80.00%** — —	No compatible expected amplicon
42	*Pichia kudriavzevii*	4909	**< 90.00%** CP021089.1 1165947 - 1165966	**< 80.00%** CP021092.1 504364 - 504383	**< 85.00%** CP021089.1 853926 - 853945	No compatible expected amplicon
43	*Candida parapsilosis*	5480	**< 85.00%** CP137570.1 1182592 - 1182611	**< 80.00%** CP137566.1 338978 - 338997	**< 85.00%** CP137572.1 981780 - 981799	No compatible expected amplicon

No.	Organism	Taxonomy ID	Forward primer match	Probe match	Reverse primer match	Compatible expected amplicon
44	*Candida tropicalis*	5482	**< 85.00%** CP047869.1 1872130 - 1872149	**< 80.00%** CP047875.1 1097406 - 1097425	**< 85.00%** CP047873.1 199060 - 199079	No compatible expected amplicon
45	*Chlamydia pneumoniae*	83558	**< 75.00%** — —	**< 80.00%** — —	**< 75.00%** — —	No compatible expected amplicon
46	*Paraclostridium bifermentans*	1490	**< 80.00%** — —	**< 80.00%** — —	**< 80.00%** — —	No compatible expected amplicon
47	*Clostridium butyricum*	1492	**< 85.00%** AP019717.1 489532 - 489551	**< 80.00%** MW548182.1 1130 - 1149	**< 75.00%** CP033246.1 140894 - 140913	No compatible expected amplicon
48	*Clostridium novyi*	1542	**< 80.00%** — —	**< 75.00%** — —	**< 80.00%** — —	No compatible expected amplicon
49	*Clostridium perfringens*	1502	**< 85.00%** AP024973.1 2685391 - 2685410	**< 85.00%** MH569437.1 1146 - 1165	**< 80.00%** CP093370.1 2120145 - 2120164	No compatible expected amplicon
50	*Clostridium sporogenes*	1509	**< 85.00%** CP009225.1 2252916 - 2252935	**< 80.00%** CP013701.1 1017139 - 1017158	**< 75.00%** CP013243.1 2897771 - 2897790	No compatible expected amplicon
51	*Corynebacterium genitalium*	38288	**< 55.00%** — —	**< 75.00%** — —	**< 50.00%** — —	No compatible expected amplicon
52	*Corynebacterium xerosis*	1725	**< 75.00%** — —	**< 80.00%** — —	**< 80.00%** — —	No compatible expected amplicon
53	*Gardnerella vaginalis*	2702	**< 85.00%** CP033836.1 574985 - 575004	**< 75.00%** CP033836.1 237096 - 237115	**< 80.00%** CP033836.1 719475 - 719494	No compatible expected amplicon
54	*Hepatitis B virus*	10407	**< 65.00%** — —	**< 75.00%** — —	**< 65.00%** — —	No compatible expected amplicon
55	*Lactobacillus crispatus*	47770	**< 85.00%** CP026503.1 1299641 - 1299660	**< 85.00%** CP114552.1 2164724 - 2164743	**< 80.00%** CP033426.1 1426862 - 1426881	No compatible expected amplicon
56	*Lactobacillus jensenii*	109790	**< 85.00%** CP018809.1 831665 - 831682	**< 80.00%** CP018809.1 580800 - 580819	**< 80.00%** CP018809.1 124765 - 124784	No compatible expected amplicon
57	*Lactobacillus delbrueckii subsp. lactis*	29397	**< 80.00%** — —	**< 80.00%** — —	**< 75.00%** — —	No compatible expected amplicon
58	*Lactiplantibacillus plantarum*	1590	**< 85.00%** CP012651.1 11867 - 11886	**< 85.00%** OR053946.1 900 - 919	**< 80.00%** CP032460.1 1090615 - 1090634	No compatible expected amplicon
59	*Limosilactobacillus reuteri*	1598	**< 85.00%** CP065540.1 1379447 - 1379466	**< 85.00%** CP014786.1 408522 - 408541	**< 80.00%** AF459437.1 2574 - 2593	No compatible expected amplicon

No.	Organism	Taxonomy ID	Forward primer match	Probe match	Reverse primer match	Compatible expected amplicon
60	*Micrococcus luteus*	1270	**< 75.00%**<br>—<br>—	**< 80.00%**<br>—<br>—	**< 75.00%**<br>—<br>—	No compatible expected amplicon
61	*Moraxella lacunata*	477	**< 70.00%**<br>—<br>—	**< 65.00%**<br>—<br>—	**< 65.00%**<br>—<br>—	No compatible expected amplicon
62	*Moraxella osloensis*	34062	**< 90.00%**<br>CP024183.1<br>50061 - 50080	**< 80.00%**<br>AP017381.1<br>127722 - 127741	**< 80.00%**<br>AP017381.1<br>1687472 - 1687491	No compatible expected amplicon
63	*Mycolicibacterium smegmatis*	1772	**< 85.00%**<br>CP009495.1<br>483528 - 483547	**< 80.00%**<br>CP009495.1<br>5227842 - 5227861	**< 80.00%**<br>CP080272.1<br>3233039 - 3233058	No compatible expected amplicon
64	*Neisseria flavescens*	484	**< 80.00%**<br>—<br>—	**< 80.00%**<br>—<br>—	**< 75.00%**<br>—<br>—	No compatible expected amplicon
65	*Pasteurella aerogenes*	749	**< 85.00%**<br>CP058953.1<br>335148 - 335167	**< 75.00%**<br>CP058953.1<br>1483972 - 1483991	**< 80.00%**<br>CP058953.1<br>701744 - 701763	No compatible expected amplicon
66	*Peptoniphilus asaccharolyticus*	1258	**< 65.00%**<br>—<br>—	**< 70.00%**<br>—<br>—	**< 60.00%**<br>—<br>—	No compatible expected amplicon
67	*Prevotella melaninogenica*	28132	**< 80.00%**<br>AP018049.1<br>271934 - 271953	**< 80.00%**<br>AP018050.1<br>601063 - 601082	**< 85.00%**<br>CP072359.1<br>334749 - 334768	No compatible expected amplicon
68	*Providencia stuartii*	588	**< 85.00%**<br>AP022374.1<br>2085288 - 2085307	**< 80.00%**<br>CP097380.1<br>139305 - 139324	**< 80.00%**<br>AP022374.1<br>3310155 - 3310174	No compatible expected amplicon
69	*Pseudomonas fluorescens*	294	**< 90.00%**<br>CP038438.1<br>320206 - 320225	**< 85.00%**<br>CP104408.1<br>3975158 - 3975177	**< 85.00%**<br>CP008896.1<br>2645460 - 2645479	No compatible expected amplicon
70	*Saccharomyces cerevisiae*	4932	**< 90.00%**<br>AP026835.1<br>769172 - 769191	**< 85.00%**<br>AP026838.1<br>503632 - 503651	**< 80.00%**<br>AP026835.1<br>667697 - 667716	No compatible expected amplicon
71	*Serratia liquefaciens*	614	**< 85.00%**<br>CP011303.1<br>3883790 - 3883809	**< 80.00%**<br>CP011303.1<br>3601217 - 3601236	**< 80.00%**<br>CP033893.1<br>436489 - 436508	No compatible expected amplicon
72	*Shigella flexneri*	623	**< 85.00%**<br>CP024470.1<br>4147825 - 4147844	**< 85.00%**<br>CP024470.1<br>4469878 - 4469897	**< 80.00%**<br>CP024470.1<br>1292189 - 1292208	No compatible expected amplicon
73	*Staphylococcus intermedius*	1285	**< 70.00%**<br>—<br>—	**< 75.00%**<br>—<br>—	**< 70.00%**<br>—<br>—	No compatible expected amplicon
74	*Streptococcus constellatus*	76860	**< 85.00%**<br>CP029207.1<br>1380044 - 1380063	**< 80.00%**<br>CP029207.1<br>619141 - 619160	**< 75.00%**<br>CP029207.1<br>1274347 - 1274366	No compatible expected amplicon

No.	Organism	Taxonomy ID	Forward primer match	Probe match	Reverse primer match	Compatible expected amplicon
75	*Streptococcus mitis*	28037	**< 80.00%**<br>—<br>—	**< 75.00%**<br>—<br>—	**< 80.00%**<br>—<br>—	No compatible expected amplicon
76	*Streptococcus parasanguinis*	1318	**< 85.00%**<br>CP134147.1<br>714397 - 714416	**< 85.00%**<br>CP133988.1<br>121858 - 121877	**< 80.00%**<br>CP133988.1<br>2118607 - 2118626	No compatible expected amplicon
77	*Clostridium haemolyticum*	84025	**< 60.00%**<br>—<br>—	**< 75.00%**<br>—<br>—	**< 50.00%**<br>—<br>—	No compatible expected amplicon
78	*Aggregatibacter actinomycetemcomitans*	714	**< 85.00%**<br>AF213680.2<br>10776 - 10795	**< 75.00%**<br>CP012958.1<br>587257 - 587276	**< 75.00%**<br>CP012958.1<br>2104520 - 2104539	No compatible expected amplicon
79	*Aeromonas hydrophila*	644	**< 90.00%**<br>CP043324.1<br>4672337 - 4672356	**< 90.00%**<br>CP027804.1<br>3123829 - 3123848	**< 85.00%**<br>AP025277.1<br>170798 - 170817	No compatible expected amplicon
80	*Alcaligenes faecalis*	511	**< 90.00%**<br>CP071957.1<br>162704 - 162723	**< 85.00%**<br>CP096916.1<br>523977 - 523996	**< 80.00%**<br>CP021883.1<br>1864737 - 1864756	No compatible expected amplicon
81	*Anaerococcus hydrogenalis*	33029	**< 65.00%**<br>—<br>—	**< 70.00%**<br>—<br>—	**< 50.00%**<br>—<br>—	No compatible expected amplicon
82	*Anaerococcus tetradius*	33036	**< 55.00%**<br>—<br>—	**< 70.00%**<br>—<br>—	**No alignment found**<br>—<br>—	No compatible expected amplicon
83	*Arcanobacterium haemolyticum*	28264	**< 80.00%**<br>—<br>—	**< 80.00%**<br>—<br>—	**< 80.00%**<br>—<br>—	No compatible expected amplicon
84	*Bacteroides fragilis*	817	**< 80.00%**<br>CP011073.1<br>1198576 - 1198595	**< 85.00%**<br>CP133097.1<br>579997 - 580016	**< 80.00%**<br>CP011073.1<br>1912995 - 1913014	No compatible expected amplicon
85	*Edwardsiella tarda*	636	**< 90.00%**<br>AP028090.1<br>1325399 - 1325418	**< 85.00%**<br>CP011359.2<br>1779160 - 1779179	**< 80.00%**<br>CP070604.1<br>2640411 - 2640430	No compatible expected amplicon
86	*Klebsiella aerogenes*	548	**< 90.00%**<br>CP055904.1<br>4653233 - 4653252	**< 85.00%**<br>CP119076.1<br>3013085 - 3013104	**< 85.00%**<br>AP022108.1<br>1157404 - 1157423	No compatible expected amplicon
87	*Klebsiella pneumoniae*	573	**< 95.00%**<br>CP025090.1<br>2147221 - 2147240	**< 90.00%**<br>CP110741.1<br>23117 - 23136	**< 85.00%**<br>CP052386.1<br>3788867 - 3788886	No compatible expected amplicon
88	*Lactobacillus delbrueckii*	1584	**< 86.36%**<br>CP045604.1<br>1206550 - 1206571	**< 80.00%**<br>CP045604.1<br>1583096 - 1583115	**< 75.00%**<br>CP049759.1<br>470500 - 470519	No compatible expected amplicon

No.	Organism	Taxonomy ID	Forward primer match	Probe match	Reverse primer match	Compatible expected amplicon
89	*Listeria monocytogenes*	1639	**< 90.00%** CP090052.1 2120822 - 2120841	**< 80.00%** CP006592.1 1760047 - 1760066	**< 80.00%** CP007583.1 935831 - 935850	No compatible expected amplicon
90	*Parvimonas micra*	33033	**< 85.00%** CP101407.1 1497359 - 1497378	**< 80.00%** CP148048.1 797106 - 797125	**< 75.00%** CP009761.1 727752 - 727771	No compatible expected amplicon
91	*Porphyromonas asaccharolytica*	28123	**< 55.00%** — —	**< 55.00%** — —	**< 60.00%** — —	No compatible expected amplicon
92	*Prevotella bivia*	28125	**< 85.00%** AP029375.1 135711 - 135730	**< 85.00%** AP029375.1 96236 - 96255	**< 80.00%** AP029375.1 816958 - 816977	No compatible expected amplicon
93	*Shigella boydii*	621	**< 90.00%** AY369140.1 1496 - 1515	**< 85.00%** CP011511.1 4525148 - 4525167	**< 80.00%** CP011511.1 3153964 - 3153983	No compatible expected amplicon
94	*Shigella sonnei*	624	**< 85.00%** CP010829.1 249374 - 249393	**< 85.00%** CP010829.1 4778677 - 4778696	**< 80.00%** CP010829.1 3203624 - 3203643	No compatible expected amplicon
95	*Streptococcus anginosus*	1328	**< 85.00%** CP118053.1 136867 - 136886	**< 80.00%** AP018548.1 1152143 - 1152162	**< 80.00%** CP068060.1 1183572 - 1183591	No compatible expected amplicon
96	*Streptococcus equinus*	1335	**< 85.71%** CP046628.1 926121 - 926140	**< 80.00%** CP076703.1 1310921 - 1310940	**< 80.00%** CP070238.1 1391030 - 1391049	No compatible expected amplicon
97	*Streptococcus intermedius*	1338	**< 85.00%** AP014880.1 1454724 - 1454743	**< 80.00%** AP014880.1 317802 - 317821	**< 75.00%** AB326213.2 5841 - 5860	No compatible expected amplicon
98	*Streptococcus oralis*	1303	**< 85.00%** CP029257.1 177493 - 177512	**< 80.00%** CP046523.1 1870011 - 1870030	**< 80.00%** CP023507.1 68145 - 68164	No compatible expected amplicon
99	*Streptococcus pneumoniae*	1313	**< 85.00%** AP018043.1 1403608 - 1403627	**< 80.00%** AP018936.1 318425 - 318444	**< 75.00%** AP018938.1 694741 - 694760	No compatible expected amplicon
100	*Streptococcus salivarius*	1304	**< 85.00%** CP009913.1 1596817 - 1596836	**< 80.00%** CP009913.1 1777595 - 1777614	**< 80.00%** CP018187.1 2152494 - 2152513	No compatible expected amplicon
101	*Vibrio cholerae*	666	**< 90.00%** AP023381.1 781793 - 781812	**< 80.00%** AP018677.1 2767748 - 2767767	**< 85.00%** AP018677.1 2976179 - 2976198	No compatible expected amplicon
102	*Yersinia enterocolitica*	630	**< 90.00%** CP107095.1 458176 - 458195	**< 85.00%** CP009367.1 513647 - 513666	**< 80.00%** CP107091.1 3053552 - 3053571	No compatible expected amplicon

No.	Organism	Taxonomy ID	Forward primer match	Probe match	Reverse primer match	Compatible expected amplicon
103	*Tannerella forsythia*	28112	**< 70.00%**<br>—<br>—	**< 65.00%**<br>—<br>—	**< 65.00%**<br>—<br>—	No compatible expected amplicon
104	*Acinetobacter schindleri*	108981	**< 85.00%**<br>CP015615.1<br>2762943 - 2762962	**< 85.00%**<br>CP025618.2<br>2559571 - 2559590	**< 80.00%**<br>CP015615.1<br>1899239 - 1899258	No compatible expected amplicon
105	*Acinetobacter ursingii*	108980	**< 80.00%**<br>AP018824.1<br>1253625 - 1253644	**< 85.00%**<br>CP089038.1<br>3445329 - 3445348	**< 80.00%**<br>AP018824.1<br>919899 - 919918	No compatible expected amplicon
106	*Aerococcus viridans*	1377	**< 85.00%**<br>CP014164.1<br>1018385 - 1018404	**< 80.00%**<br>CP014164.1<br>871510 - 871529	**< 75.00%**<br>CP014164.1<br>1150743 - 1150762	No compatible expected amplicon
107	*Corynebacterium glutamicum*	1718	**< 80.00%**<br>CP041728.1<br>222400 - 222419	**< 90.00%**<br>AP017557.2<br>794455 - 794474	**< 80.00%**<br>AF434799.1<br>199 - 218	No compatible expected amplicon
108	*Lactobacillus gasseri*	1596	**< 85.00%**<br>CP071801.1<br>1554553 - 1554572	**< 80.00%**<br>CP054875.1<br>1499046 - 1499065	**< 80.00%**<br>CP071801.1<br>1106708 - 1106727	No compatible expected amplicon
109	*Megasphaera elsdenii*	907	**< 85.00%**<br>CP027569.1<br>2465527 - 2465546	**< 85.00%**<br>KX130890.1<br>1178 - 1197	**< 75.00%**<br>CP027569.1<br>1781396 - 1781415	No compatible expected amplicon
110	*Peptostreptococcus anaerobius*	1261	**< 60.00%**<br>—<br>—	**< 75.00%**<br>—<br>—	**< 65.00%**<br>—<br>—	No compatible expected amplicon
111	*Staphylococcus delphini*	53344	**< 80.00%**<br>—<br>—	**< 75.00%**<br>—<br>—	**< 80.00%**<br>—<br>—	No compatible expected amplicon
112	*Staphylococcus gallinarum*	1293	**< 85.00%**<br>CP069082.1<br>2732852 - 2732871	**< 85.00%**<br>MN904855.1<br>1176 - 1195	**< 75.00%**<br>CP069082.1<br>688400 - 688419	No compatible expected amplicon
113	*Neisseria meningitidis*	487	**< 100.00%**<br>AY682396.1<br>137 - 156	**< 95.00%**<br>AY682396.1<br>188 - 207	**< 75.00%**<br>AP024489.1<br>1301837 - 1301856	No compatible expected amplicon
114	*Chlamydia trachomatis*	813	**< 85.00%**<br>CP006671.1<br>524402 - 524421	**< 70.00%**<br>CP006671.1<br>773241 - 773260	**< 70.00%**<br>CP006671.1<br>543426 - 543445	No compatible expected amplicon

Each oligonucleotide-match cell lists, **from top to bottom, the highest reported sequence identity, the corresponding highest-homology accession, and the sequence position**. An em dash (—) indicates that the source report did not provide accession or position details. None of the 114 non-target organisms showed a compatible arrangement of forward- and reverse-primer binding sites with an intervening probe-binding site capable of generating the expected amplicon (<1 kb). The analysis used NCBI sequence records available as of 10 February 2025.

Supplementary Table S4. Validation results for 100 clinical samples.

#	Sample number	Type	Diagnosis	Ct value	Result
1	SD-CBU001	Urine	*Neisseria gonorrhoeae*	38.47	NG+
2	SD-CBU002	Urine	*Neisseria gonorrhoeae*	31.44	NG+
3	SD-CBU003	Urine	*Neisseria gonorrhoeae*	29.33	NG+
4	SD-CBU004	Urine	*Neisseria gonorrhoeae*	33.09	NG+
5	SD-CBU006	Urine	*Neisseria gonorrhoeae*	39.87	NG+
6	SD-CBU007	Urine	*Neisseria gonorrhoeae*	34.84	NG+
7	SD-CBU008	Urine	*Neisseria gonorrhoeae*	33.12	NG+
8	SD-CBU015	Urine	*Neisseria gonorrhoeae*	31.79	NG+
9	SD-CBU016	Urine	*Neisseria gonorrhoeae*	33.63	NG+
10	SD-CBU017	Urine	*Neisseria gonorrhoeae*	29.58	NG+
11	SD-CBU018	Urine	*Neisseria gonorrhoeae*	30.02	NG+
12	SD-CBU020	Urine	*Neisseria gonorrhoeae*	36.51	NG+
13	SD-CBU021	Urine	*Neisseria gonorrhoeae*	31.12	NG+
14	SD-CBU022	Urine	*Neisseria gonorrhoeae*	34.80	NG+
15	SD-CBU023	Urine	*Neisseria gonorrhoeae*	32.88	NG+
16	SD-CBU024	Urine	*Neisseria gonorrhoeae*	31.79	NG+
17	SD-CBU025	Urine	*Neisseria gonorrhoeae*	33.35	NG+
18	SD-CBU026	Urine	*Neisseria gonorrhoeae*	35.49	NG+
19	SD-CBU027	Urine	*Neisseria gonorrhoeae*	34.60	NG+
20	SD-CBU028	Urine	*Neisseria gonorrhoeae*	35.63	NG+
21	SD-CBU029	Urine	*Neisseria gonorrhoeae*	27.43	NG+
22	SD-CBU030	Urine	*Neisseria gonorrhoeae*	36.01	NG+
23	SD-CBU031	Urine	*Neisseria gonorrhoeae*	27.66	NG+
24	SD-CBU034	Urine	*Neisseria gonorrhoeae*	30.88	NG+
25	SD-CBU036	Urine	*Neisseria gonorrhoeae*	35.30	NG+
26	SD-CBU038	Urine	*Neisseria gonorrhoeae*	31.28	NG+
27	SD-CBU039	Urine	*Neisseria gonorrhoeae*	32.10	NG+

28	SD-CBU040	Urine	*Neisseria gonorrhoeae*	29.31	NG+
29	SD-CBU041	Urine	*Neisseria gonorrhoeae*	25.52	NG+
30	SD-CBU044	Urine	*Neisseria gonorrhoeae*	28.06	NG+
31	SD-CBU045	Urine	*Neisseria gonorrhoeae*	28.41	NG+
32	SD-CBU046	Urine	*Neisseria gonorrhoeae*	31.05	NG+
33	SD-CBU047	Urine	*Neisseria gonorrhoeae*	30.82	NG+
34	SD-CBU056	Urine	*Neisseria gonorrhoeae*	27.47	NG+
35	SD-CBU057	Urine	*Neisseria gonorrhoeae*	31.83	NG+
36	SD-MAU001	Urine	*Neisseria gonorrhoeae*	33.22	NG+
37	SD-MAU004	Urine	*Neisseria gonorrhoeae*	28.66	NG+
38	SD-MAU005	Urine	*Neisseria gonorrhoeae*	30.43	NG+
39	SD-MAU007	Urine	*Neisseria gonorrhoeae*	35.07	NG+
40	SD-MAU010	Urine	*Neisseria gonorrhoeae*	28.42	NG+
41	SD-CBV001	Vaginal Swab	*Neisseria gonorrhoeae*	29.64	NG+
42	SD-CBV002	Vaginal Swab	*Neisseria gonorrhoeae*	24.32	NG+
43	SD-CBV003	Vaginal Swab	*Neisseria gonorrhoeae*	24.74	NG+
44	SD-CBV004	Vaginal Swab	*Neisseria gonorrhoeae*	20.89	NG+
45	SD-CBV005	Vaginal Swab	*Neisseria gonorrhoeae*	29.93	NG+
46	SD-CBV006	Vaginal Swab	*Neisseria gonorrhoeae*	27.05	NG+
47	SD-CBV007	Vaginal Swab	*Neisseria gonorrhoeae*	27.07	NG+
48	SD-CBV009	Vaginal Swab	*Neisseria gonorrhoeae*	22.26	NG+
49	SD-CBV010	Vaginal Swab	*Neisseria gonorrhoeae*	30.11	NG+
50	SD-CBV011	Vaginal Swab	*Neisseria gonorrhoeae*	29.86	NG+
51	SD-CBV012	Vaginal Swab	*Neisseria gonorrhoeae*	26.05	NG+
52	SD-CBV013	Vaginal Swab	*Neisseria gonorrhoeae*	23.27	NG+
53	SD-CBV014	Vaginal Swab	*Neisseria gonorrhoeae*	25.25	NG+
54	SD-CBV015	Vaginal Swab	*Neisseria gonorrhoeae*	21.19	NG+
55	SD-CBV017	Vaginal Swab	*Neisseria gonorrhoeae*	26.44	NG+
56	SD-CBV018	Vaginal Swab	*Neisseria gonorrhoeae*	23.22	NG+

57	SD-CCV001	Vaginal Swab	*Neisseria gonorrhoeae*	28.59	NG+
58	SD-CCV003	Vaginal Swab	*Neisseria gonorrhoeae*	24.63	NG+
59	SD-CCV006	Vaginal Swab	*Neisseria gonorrhoeae*	29.81	NG+
60	SD-CCV008	Vaginal Swab	*Neisseria gonorrhoeae*	28.77	NG+
61	SD-CCV009	Vaginal Swab	*Neisseria gonorrhoeae*	30.38	NG+
62	SD-CCV010	Vaginal Swab	*Neisseria gonorrhoeae*	24.50	NG+
63	SD-CCV011	Vaginal Swab	*Neisseria gonorrhoeae*	29.06	NG+
64	SD-MAV002	Vaginal Swab	*Neisseria gonorrhoeae*	23.40	NG+
65	SD-MAV003	Vaginal Swab	*Neisseria gonorrhoeae*	22.03	NG+
66	SD-MAV005	Vaginal Swab	*Neisseria gonorrhoeae*	27.55	NG+
67	SD-MAV007	Vaginal Swab	*Neisseria gonorrhoeae*	26.96	NG+
68	SD-MAV009	Vaginal Swab	*Neisseria gonorrhoeae*	28.07	NG+
69	SD-MAV018	Vaginal Swab	*Neisseria gonorrhoeae*	29.54	NG+
70	SD-MAV020	Vaginal Swab	*Neisseria gonorrhoeae*	24.77	NG+
71	SD-MAV026	Vaginal Swab	*Neisseria gonorrhoeae*	24.64	NG+
72	SD-MAV027	Vaginal Swab	*Neisseria gonorrhoeae*	25.52	NG+
73	SD-MAV028	Vaginal Swab	*Neisseria gonorrhoeae*	26.61	NG+
74	SD-MAV029	Vaginal Swab	*Neisseria gonorrhoeae*	29.42	NG+
75	SD-MAV031	Vaginal Swab	*Neisseria gonorrhoeae*	28.96	NG+
76	SD-MAV032	Vaginal Swab	*Neisseria gonorrhoeae*	24.77	NG+
77	SD-MAV033	Vaginal Swab	*Neisseria gonorrhoeae*	27.09	NG+
78	SD-MAV034	Vaginal Swab	*Neisseria gonorrhoeae*	22.81	NG+
79	SD-MAV035	Vaginal Swab	*Neisseria gonorrhoeae*	23.28	NG+
80	SD-MAV040	Vaginal Swab	*Neisseria gonorrhoeae*	26.11	NG+
81	SD-CBU121	Urine	Negative	None	Negative
82	SD-CBU122	Urine	Negative	None	Negative
83	SD-CBU123	Urine	Negative	None	Negative
84	SD-CBU124	Urine	Negative	None	Negative
85	SD-CBU125	Urine	Negative	None	Negative

86	SD-CBU126	Urine	Negative	None	Negative
87	SD-CBU127	Urine	Negative	None	Negative
88	SD-CBU128	Urine	Negative	None	Negative
89	SD-CBU129	Urine	Negative	None	Negative
90	SD-CBU130	Urine	Negative	None	Negative
91	SD-CBV166	Vaginal Swab	Negative	None	Negative
92	SD-CBV167	Vaginal Swab	Negative	None	Negative
93	SD-CBV168	Vaginal Swab	Negative	None	Negative
94	SD-CBV169	Vaginal Swab	Negative	None	Negative
95	SD-CBV170	Vaginal Swab	Negative	None	Negative
96	SD-CBV171	Vaginal Swab	Negative	None	Negative
97	SD-CBV172	Vaginal Swab	Negative	None	Negative
98	SD-CBV173	Vaginal Swab	Negative	None	Negative
99	SD-CBV174	Vaginal Swab	Negative	None	Negative
100	SD-CBV175	Vaginal Swab	Negative	None	Negative

Supplementary Table S5. Specimen-level paired qualitative comparison between the developed *porA*-targeted qPCR assay and Cobas 4800 CT/NG testing.

#	Sample number	Type	Diagnosis	Cobas 4800 CT/NG result	Developed qPCR result	Concordance
1	SD-CAU041	Urine	*Neisseria gonorrhoeae*	NG+	NG+	Concordant
2	SD-CAU043	Urine	*Neisseria gonorrhoeae*	NG+	NG+	Concordant
3	SD-CAU044	Urine	*Neisseria gonorrhoeae*	NG+	NG+	Concordant
4	SD-CAU045	Urine	*Neisseria gonorrhoeae*	NG+	NG+	Concordant
5	SD-CAU047	Urine	*Neisseria gonorrhoeae*	NG+	NG+	Concordant
6	SD-CAU048	Urine	*Neisseria gonorrhoeae*	NG+	NG+	Concordant
7	SD-CAU050	Urine	*Neisseria gonorrhoeae*	NG+	NG+	Concordant
8	SD-CAU052	Urine	*Neisseria gonorrhoeae*	NG+	NG+	Concordant
9	SD-CAU053	Urine	*Neisseria gonorrhoeae*	NG+	NG+	Concordant
10	SD-CAU054	Urine	*Neisseria gonorrhoeae*	NG+	NG+	Concordant
11	SD-CAU055	Urine	*Neisseria gonorrhoeae*	NG+	NG+	Concordant
12	SD-CAU056	Urine	*Neisseria gonorrhoeae*	NG+	NG+	Concordant
13	SD-CAU058	Urine	*Neisseria gonorrhoeae*	NG+	NG+	Concordant
14	SD-CAU059	Urine	*Neisseria gonorrhoeae*	NG+	NG+	Concordant
15	SD-CAU060	Urine	*Neisseria gonorrhoeae*	NG+	NG+	Concordant
16	SD-CAU061	Urine	*Neisseria gonorrhoeae*	NG+	NG+	Concordant
17	SD-CAU062	Urine	*Neisseria gonorrhoeae*	NG+	NG+	Concordant
18	SD-CAU065	Urine	*Neisseria gonorrhoeae*	NG+	NG+	Concordant
19	SD-CAU066	Urine	*Neisseria gonorrhoeae*	NG+	NG+	Concordant
20	SD-CAU068	Urine	*Neisseria gonorrhoeae*	NG+	NG+	Concordant
21	SD-CAU069	Urine	*Neisseria gonorrhoeae*	NG+	NG+	Concordant
22	SD-CAU070	Urine	*Neisseria gonorrhoeae*	NG+	NG+	Concordant
23	SD-CAU071	Urine	*Neisseria gonorrhoeae*	NG+	NG+	Concordant
24	SD-CAU072	Urine	*Neisseria gonorrhoeae*	NG+	NG+	Concordant
25	SD-CAU074	Urine	*Neisseria gonorrhoeae*	NG+	NG+	Concordant

26	SD-CAU075	Urine	*Neisseria gonorrhoeae*	NG+	NG+	Concordant
27	SD-CAU076	Urine	*Neisseria gonorrhoeae*	NG+	NG+	Concordant
28	SD-CAU077	Urine	*Neisseria gonorrhoeae*	NG+	NG+	Concordant
29	SD-CAU078	Urine	*Neisseria gonorrhoeae*	NG+	NG+	Concordant
30	SD-CAU079	Urine	*Neisseria gonorrhoeae*	NG+	NG+	Concordant
31	SD-CAU001	Urine	Negative	Negative	Negative	Concordant
32	SD-CAU002	Urine	Negative	Negative	Negative	Concordant
33	SD-CAU003	Urine	Negative	Negative	Negative	Concordant
34	SD-CAU004	Urine	Negative	Negative	Negative	Concordant
35	SD-CAU005	Urine	Negative	Negative	Negative	Concordant
36	SD-CAU006	Urine	Negative	Negative	Negative	Concordant
37	SD-CAU007	Urine	Negative	Negative	Negative	Concordant
38	SD-CAU008	Urine	Negative	Negative	Negative	Concordant
39	SD-CAU009	Urine	Negative	Negative	Negative	Concordant
40	SD-CAU010	Urine	Negative	Negative	Negative	Concordant
41	SD-CAU011	Urine	Negative	Negative	Negative	Concordant
42	SD-CAU012	Urine	Negative	Negative	Negative	Concordant
43	SD-CAU013	Urine	Negative	Negative	Negative	Concordant
44	SD-CAU014	Urine	Negative	Negative	Negative	Concordant
45	SD-CAU015	Urine	Negative	Negative	Negative	Concordant
46	SD-CAU016	Urine	Negative	Negative	Negative	Concordant
47	SD-CAU017	Urine	Negative	Negative	Negative	Concordant
48	SD-CAU018	Urine	Negative	Negative	Negative	Concordant
49	SD-CAU019	Urine	Negative	Negative	Negative	Concordant
50	SD-CAU020	Urine	Negative	Negative	Negative	Concordant
51	SD-CAU021	Urine	Negative	Negative	Negative	Concordant
52	SD-CAU022	Urine	Negative	Negative	Negative	Concordant
53	SD-CAU023	Urine	Negative	Negative	Negative	Concordant
54	SD-CAU024	Urine	Negative	Negative	Negative	Concordant

55	SD-CAU025	Urine	Negative	Negative	Negative	Concordant
56	SD-CAU026	Urine	Negative	Negative	Negative	Concordant
57	SD-CAU027	Urine	Negative	Negative	Negative	Concordant
58	SD-CAU028	Urine	Negative	Negative	Negative	Concordant
59	SD-CAU029	Urine	Negative	Negative	Negative	Concordant
60	SD-CAU030	Urine	Negative	Negative	Negative	Concordant
61	SD-CAU031	Urine	Negative	Negative	Negative	Concordant
62	SD-CAU032	Urine	Negative	Negative	Negative	Concordant
63	SD-CAU033	Urine	Negative	Negative	Negative	Concordant
64	SD-CAU034	Urine	Negative	Negative	Negative	Concordant
65	SD-CAU035	Urine	Negative	Negative	Negative	Concordant
66	SD-CAU036	Urine	Negative	Negative	Negative	Concordant
67	SD-CAU037	Urine	Negative	Negative	Negative	Concordant
68	SD-CAU038	Urine	Negative	Negative	Negative	Concordant
69	SD-CAU039	Urine	Negative	Negative	Negative	Concordant
70	SD-CAU040	Urine	Negative	Negative	Negative	Concordant

Supplementary Table S6. Individual Ct values and summary statistics for the Z017-based standard curve generated from triplicate qPCR measurements.

Z017 concentration (CFU/mL)	Ct, replicate 1	Ct, replicate 2	Ct, replicate 3	Mean Ct	SD	CV (%)
1.00×10^7	17.20	17.59	17.59	17.46	0.23	1.29
1.00×10^6	20.99	20.83	20.67	20.83	0.16	0.77
1.00×10^5	24.67	24.57	24.42	24.55	0.13	0.51
1.00×10^4	28.13	28.01	27.95	28.03	0.09	0.33
1.00×10^3	31.07	31.82	31.93	31.61	0.47	1.48
1.00×10^2	34.66	34.70	34.19	34.52	0.28	0.82
1.00×10^1	None	None	None	N/A*	N/A	N/A

*N/A: not applicable

Supplementary Table S7. Contextual comparison of analytical performance metrics reported for selected published *Neisseria gonorrhoeae* detection assays.

Study	Assay type	Reported analytical metric	Present-study value expressed using the corresponding parameter	Interpretation
Present study	2-step *porA*-targeted TaqMan qPCR	1×10^{-5} ng/μL; 1 μL *N. gonorrhoeae* DNA input	0.01 pg/reaction; approximately 4.1 genome equivalents/reaction	Lowest input detected in the original serial-dilution experiment
Aitlhaj-Mhand *et al.* [29]	*porA*-targeted real-time PCR	0.6 pg *N. gonorrhoeae* DNA	Present study: 0.01 pg/reaction	Both values are presented as DNA-mass inputs; direct performance ranking was not performed
Ahmadi *et al.* [30]	LAMP	72 copies/reaction	Present study: approximately 4.1 genome equivalents/reaction	Both values are expressed per analytical reaction; genome-equivalent conversion for the present study is theoretical
Chen *et al.* [31]	Thio-NAD cycling ELISA	1.1×10^4 ~ 1.8×10^4 CFU/mL	Not directly comparable; the present study detected 0.01 pg DNA per reaction (approximately 4.1 theoretical genome equivalents per reaction)	Chen *et al.* reported CFU/mL using a protein-based ELISA, whereas the present study reports post-extraction DNA input per reaction using qPCR. Because the measurement principles, sample processing, and reporting units differ, no direct unit conversion or analytical-performance ranking was performed.

The present-study genome-equivalent value was calculated using an approximate 2.2 Mb *N. gonorrhoeae* genome and an estimated genome mass of approximately 2.4 fg. These values are provided for descriptive contextualization only and should not be interpreted as a direct analytical sensitivity ranking across studies.

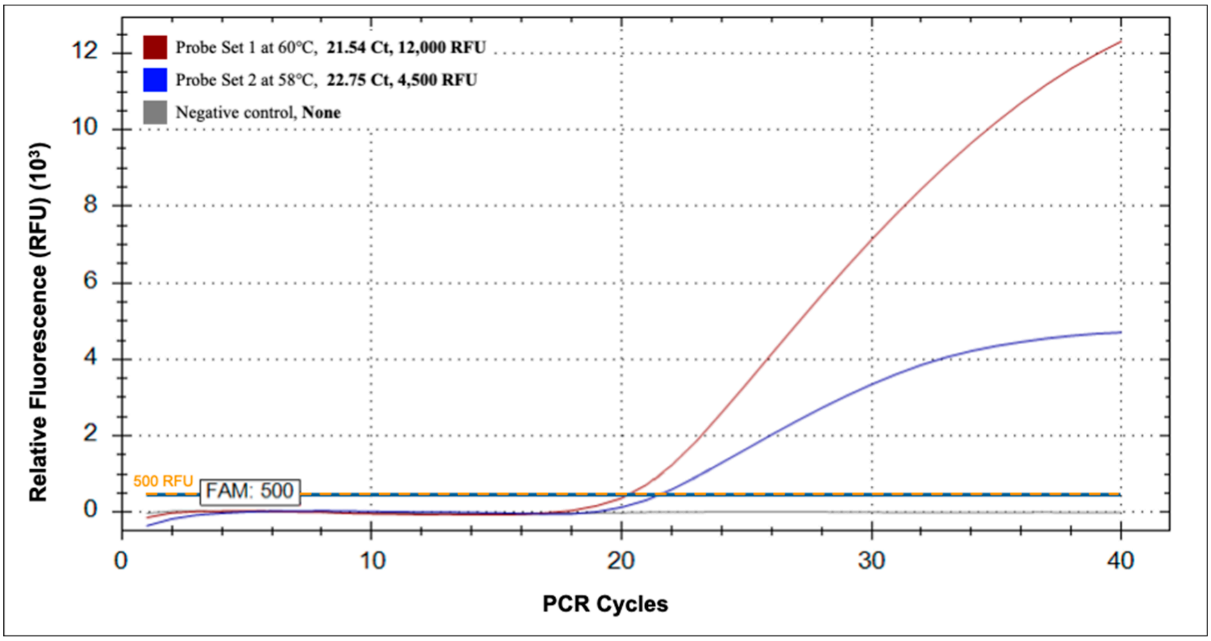

Supplementary Figure S1. Preliminary amplification curves of two candidate *porA* probe sets for *Neisseria gonorrhoeae* detection. Candidate set 1 was evaluated using *N. gonorrhoeae* ATCC 19424 DNA with a 60 °C annealing/extension condition, whereas candidate set 2 was evaluated using the same DNA template with a 58 °C annealing/extension condition. Nuclease-free water was used as the no-template control. The curves are shown for preliminary screening under their respective conditions and should not be interpreted as a fully matched head-to-head comparison.

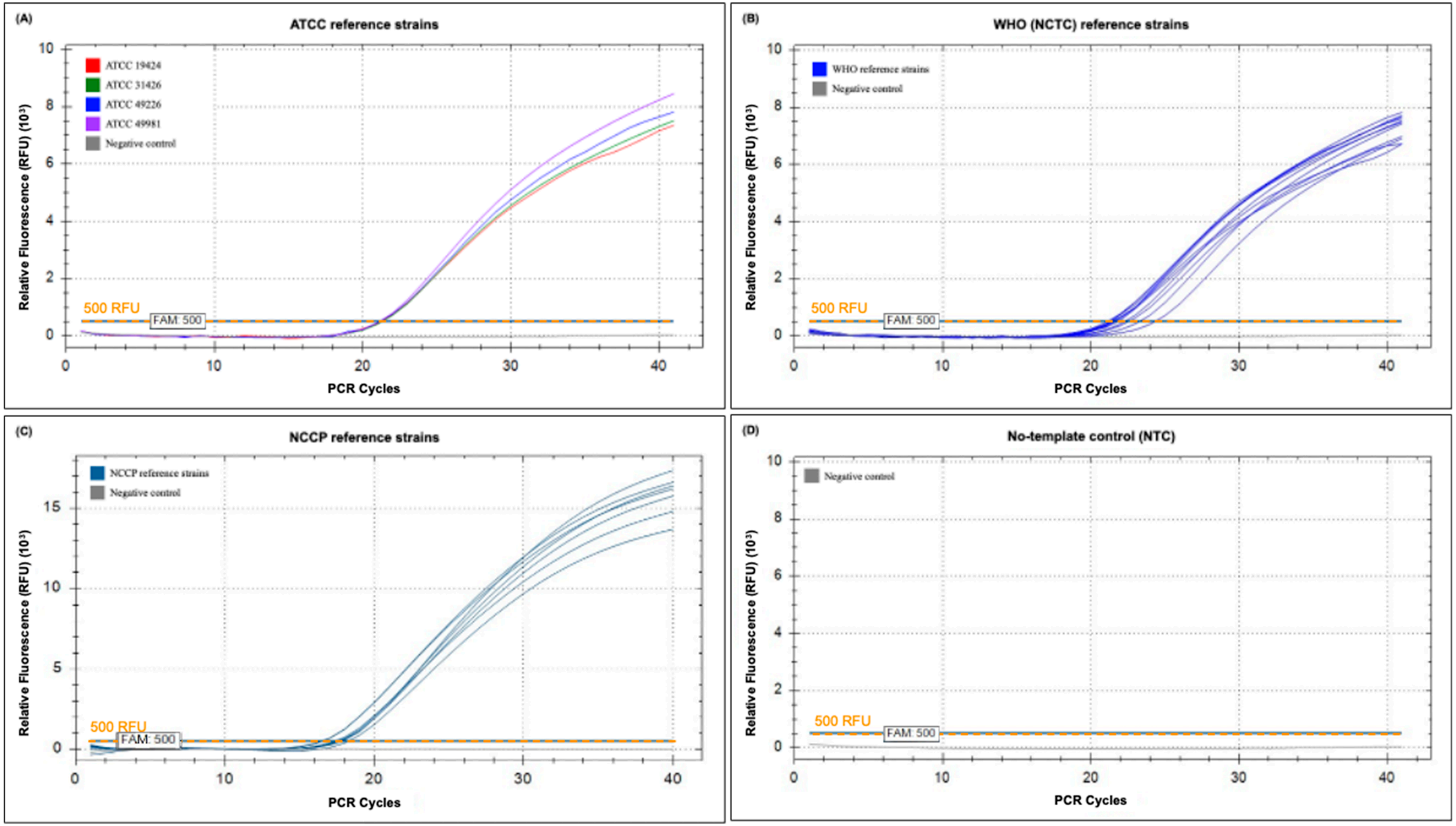

Supplementary Figure S2. Representative amplification curves obtained during validation of the final *porA*-targeted TaqMan

qPCR assay conditions using 23 *Neisseria gonorrhoeae* reference strains. (A) ATCC reference strains: ATCC 19424, ATCC 31426, ATCC 49226, and ATCC 49981. (B) WHO (NCTC) reference strains: NCTC 13477, NCTC 13478, NCTC 13479, NCTC 13480, NCTC 13481, NCTC 13482, NCTC 13483, NCTC 13484, NCTC 14208, NCTC 15801, NCTC 15802, and NCTC 15803. (C) NCCP reference strains: NCCP 11343, NCCP 11351, NCCP 11363, NCCP 11395, NCCP 11937, NCCP 16057, and NCCP 16058. (D) Negative control: No-template control (NTC; nuclease-free water). A fixed fluorescence threshold of 500 RFU was applied. All tested *N. gonorrhoeae* reference strains crossed the threshold within 40 cycles, whereas the NTC did not cross the threshold during the 40-cycle protocol.

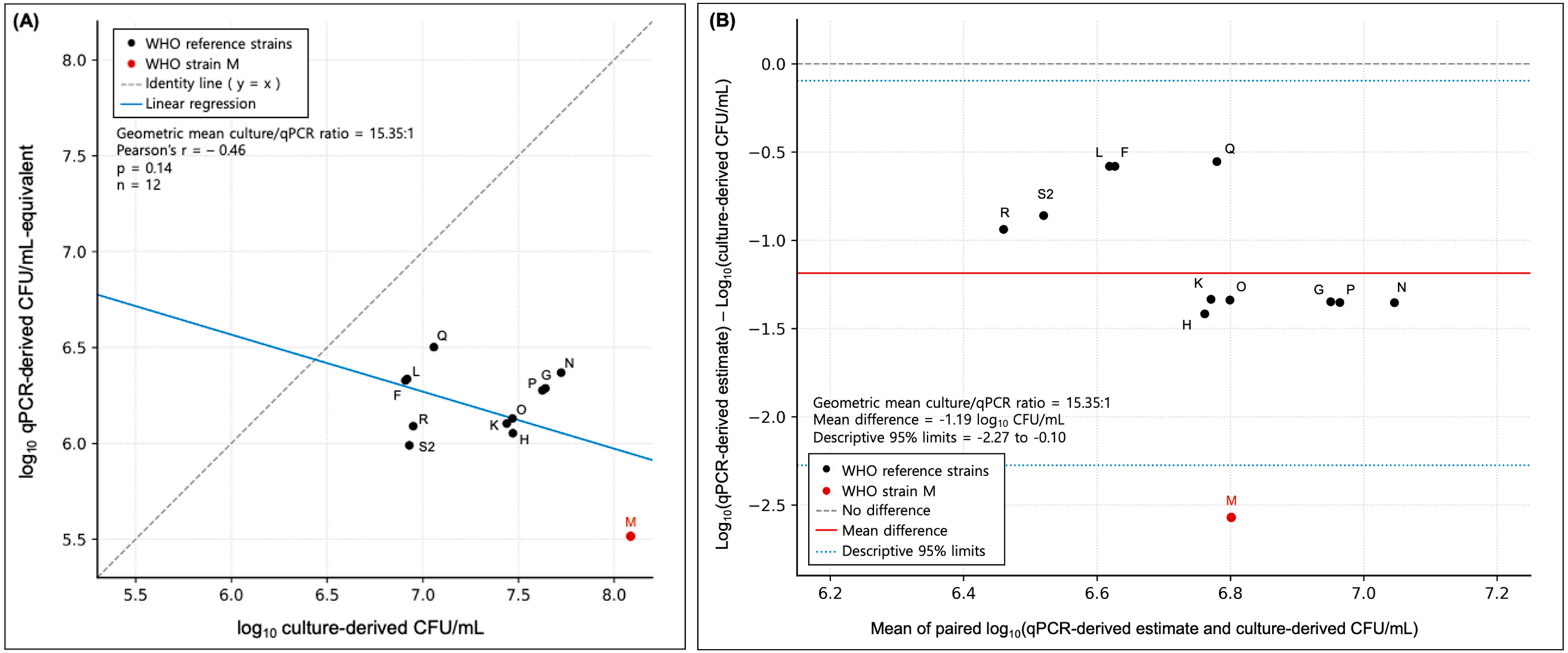

Supplementary Figure S3. Descriptive comparison of Z017-calibrated qPCR-derived CFU/mL-equivalent estimates and culture-derived CFU/mL values for 12 cultured WHO reference strains. (A) Scatter plot of $\log_{10}$-transformed culture-derived CFU/mL values versus $\log_{10}$-transformed qPCR-derived CFU/mL-equivalent estimates. Each point represents one WHO reference strain; letters indicate the corresponding WHO strain designation. The blue solid line indicates the linear regression fit, and the gray

dashed line indicates the identity line (y = x). The paired values were not significantly correlated (Pearson's r = −0.46, p = 0.14; n = 12). The geometric mean culture/qPCR ratio was 15.35:1. (B) Descriptive paired $\log_{10}$-difference plot. The y-axis represents $\log_{10}$ (qPCR-derived CFU/mL-equivalent estimate) − $\log_{10}$ (culture-derived CFU/mL). The red solid line indicates the mean paired difference (−1.19 $\log_{10}$ CFU/mL), the blue dotted lines indicate the descriptive 95% limits (−2.27 to −0.10 $\log_{10}$ CFU/mL), and the gray dashed line indicates no difference. The red point denotes WHO strain M, which showed the largest culture/qPCR discrepancy. These analyses are descriptive and exploratory and do not establish direct interchangeability between qPCR-derived molecular estimates and culture-derived viable CFU/mL values.
